# Signature of the Paleo-Course Changes in the São Francisco River as Source of Genetic Structure in Neotropical *Pithecopus nordestinus* (Phyllomedusinae, Anura) Treefrog

**DOI:** 10.3389/fgene.2019.00728

**Published:** 2019-08-14

**Authors:** Daniel Pacheco Bruschi, Elen Arroyo Peres, Luciana Bolsoni Lourenço, Luiz Filipe de Macedo Bartoleti, Thadeu Sobral-Souza, Shirlei Maria Recco-Pimentel

**Affiliations:** ^1^Department of Genetics, University of Parana (UFPR), Curitiba, Brazil; ^2^Department of Zoology, Institute of Biosciences, University of São Paulo, São Paulo, Brazil; ^3^Department of Structural and Functional Biology, University of Campinas (UNICAMP), Campinas, Brazil; ^4^Department of Genetics and Evolution and Bioagent, University of Campinas (UNICAMP); Campinas, Brazil; ^5^Spatial Ecology and Conservation Lab (LEEC), São Paulo State University (UNESP), Rio Claro, São Paulo, Brazil

**Keywords:** Anura, vicariance, Riverine barrier hypothesis, phylogeographic break, allopatric diversification

## Abstract

Historical processes that have interrupted gene flow between distinct evolutionary lineages have played a fundamental role in the evolution of the enormous diversity of species found in the Neotropical region. Numerous studies have discussed the role of geographic barriers and Pleistocene forest refugia in the diversification of the region’s biodiversity. In the present study, we investigated the relative contribution of these different factors to the evolutionary history of *Pithecopus nordestinus*, a Neotropical tree frog, which is amply distributed in the Brazilian Atlantic Forest and adjacent areas of the Caatinga biome. We used an extensive sample and multilocus DNA sequences to provide an overview of the intraspecific genetic diversity of *P. nordestinus*, characterize historical diversification patterns, and identify possible phylogenetic splits. We tested different scenarios of diversification based on Pleistocene Refugia and river barrier models using approximate Bayesian computation (ABC) and ecological niche modeling (ENM). The phylogenetic approach indicate the occurrence of processes of phylogeographic divergence in both time and space, related to historical shifts in the course of the São Francisco River during Plio-Pleistocene period, resulting in two principal, highly divergent clades. The ABC model provided strong statistical support for this scenario, confirming the hypothesis that the São Francisco River acted as an effective geographical barrier during vicariant events in the evolutionary history of *P. nordestinus*. We believe that the climatic changes that occurred during the Pleistocene also played a secondary role in the genetic signatures identified, reinforcing the divergence of populations isolated by physical barriers. These findings reinforce the conclusion that the two models of diversification (geographic barriers and refugia) are not mutually exclusive in the Neotropical domain but may interact extensively during the diversification of species on a regional scale.

## Introduction

Models of speciation are widely invoked to explain the origin and diversification of lineages and species complexes in the Brazilian Atlantic Forest, a global hotspot. As the Atlantic Forest presents high species richness, endemism, different phytophisionomies, and a complex topographic landscape (Turchetto-Zolet et al., 2013), it has long been a target for the investigation of the evolutionary processes that promote lineage divergences in several taxa (Carnaval et al., 2009; Thomé et al., 2014; Álvarez-Presas et al., 2014; Magalhães et al., 2014; Peres et al., 2018).

A range of organisms present high geographically associated genetic structure in different areas of Atlantic Forest, reflecting historical discontinuities in gene flow (Álvarez-Presas et al., 2014; Menezes et al., 2016; Frantini-Silva et al., 2017); however, the origin of these allopatric processes remains unclear. Two main sources of vicariance have been proposed: i) the isolation of populations and reduction of gene flow through the formation of geomorphological barriers (Amaral et al., 2013; Thomé et al., 2014; Cazé et al., 2016; Peres et al., 2018) and ii) habitat fragmentation resulting from historical climatic changes during the (Pleistocene Refuges Hypothesis; Haffer, 1969; Vanzolini and Williams, 1981), restricting gene flow among fragments (Carnaval et al., 2009; Menezes et al., 2016).

Phylogeographic studies with different taxa report genetic breaks spatial or temporally congruent with specific geomorphological features of the Atlantic Forest (as rivers or mountain ranges), which have been highlighted to explain intra- and/or interspecific diversification of lizards (e.g., Werneck et al., 2015), rodents (Nascimento et al., 2011; Nascimento et al., 2013), amphibians (Thomé et al., 2010), plants (Cazé et al., 2016), harvestmen (Peres et al., 2018), and many other groups. In several lowland species, the phylogeographic patterns can be explained by the Riverine Barrier hypothesis, initially proposed by Wallace (1852), which establishes that rivers are effective barriers to gene flow between populations on opposite margins, resulting in their isolation and genetic differentiation along time (Amaral et al., 2013; Cazé et al., 2016). According to this hypothesis’ predictions, lower levels of genetic differentiation would be expected among populations from the same margin, species from opposite river margins would be typically sister taxa, and the timing of divergence events would coincide with the geological changes in the landscape (Moritz et al., 2000).

The genetic signatures associated with Pleistocene climate events, by contrast, would reflect the influence of forest relict during the Last Glacial Maximum [LGM; ca. 21,000 years ago (kya)] on allopatric diversification, as observed in many taxa (Carnaval et al., 2009; Cabanne et al., 2016; Menezes et al., 2016). Thus, if the diversification of lineages was determined by Pleistocene climate changes, the divergence times would be expected to coincide with this period; in addition, higher genetic diversity would be found in populations from areas presumed to be climatically stable (refuges), contrasting with the lower diversity in populations outside these putative refuges, which would be consistent with a genetic signature of recent demographic expansion (Carnaval et al., 2009). However, the same episode of climate shifts could impact differently the molecular diversity of organisms due to the dispersal model, demographic profile, or generation time of the species (Arenas et al., 2012; Arenas et al., 2014). Thus, to recognize the genomic signature of these events request an evaluation under species-specific optical (Mona et al., 2014).

**Figure 1:**
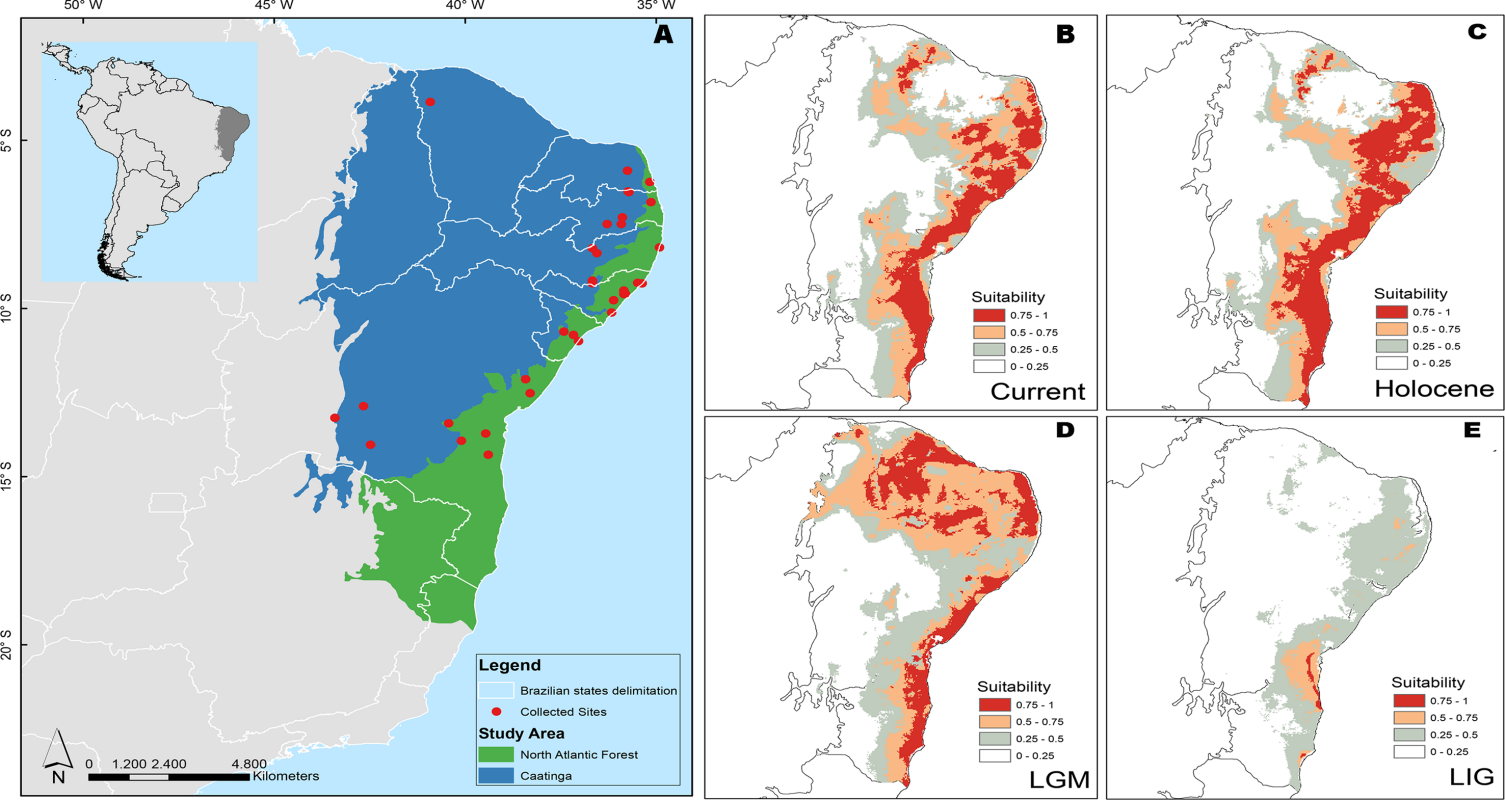
Distribution of morphoclimatic domains and sampling localities in Brazil for specimens of *P. nordestinus* included in this study **(A)** and Ecological Niche Modeling (ENM) showing suitable areas for the *P. nordestinus* potential occurrence in present and past climate scenarios. ENM prediction for present day **(B)**, during 6 ka–mid-Holocene (HOL) **(C)**; 21 ka–Last Glacial Maximum (LGM) **(D)**; and 120 ka–Last Interglacial Maximum (LIG) **(E)**. See Material and Methods for more details.

The anuran *Pithecopus nordestinus* (Anura, Arboranae, Phyllomedusidae) is widely distributed in the Atlantic Forest and adjacent areas of Caatinga scrublands (Frost, 2019). This species represents an interesting model to test hypotheses on the respective roles of geographical barriers and forest retraction during the LGM as vicariant determinants of lineage diversification. The phylogenetic hypothesis proposed by Faivovich et al. (2010) for the Phyllomedusinae family identified divergence in the gene sequences of three widely dispersed populations of the *P. nordestinus*, with uncorrected pairwise distances ranging up to 10.4%. This intraspecific diversity was spatially structured, since the most divergent populations were those from Passos do Camaragibe (Alagoas state) and Maracás (Bahia), the type locality of *P. nordestinus* (Faivovich et al., 2010). This geographical association of *P. nordestinus* sequences is consistent with the São Francisco River (SFR) delimitation, recognized as one of the main physical barriers to the dispersion of the biota in the northern Atlantic Forest. The SFR is the second largest river system in Brazil (**Figure 1**). Its lowest stretch separates the Brazilian states of Alagoas and Sergipe and is assumed to play an important role in the phylogeographic patterns of a number of different organisms (Siedchlag et al., 2010; Werneck et al., 2012; Nascimento et al., 2013). However, the pattern of genetic divergence described by Faivovich et al. (2010) also coincides with the distribution of the two northernmost Atlantic Forest Pleistocene refugia, the Pernambuco and Bahia centers of endemism (Carnaval and Moritz, 2008). The Pernambuco center is located in the northernmost portion of the Atlantic Forest, and it is separated from the Bahia center by the São Francisco River (Carnaval and Moritz, 2008).

To test the hypotheses on the possible historical scenarios that shaped the genetic diversity of this species, we sampled *P. nordestinus* throughout most of its geographical distribution to obtain phylogenetic inferences, genetic diversity indices, demographic patterns, and divergence times. We modeled the species potential distribution area in the present and in the past, and based on the historical species distribution and the main lineages recovered in our analyses (see Results), we designed and evaluated two biogeographic scenarios using coalescent simulations, as follows: i) SFR as a barrier to gene flow between ancient interbreeding populations currently located in opposite margins (resulting in allopatric speciation) and ii) ancient lineages diversified during Pleistocene climate shifts that created refugia, bisected populations.

## Material and Methods

### Study Region and Collection of Samples

(**Figure 1A**; **Table S1A**) of the *P. nordestinus* species. These samples covered ∼90% of the combined distribution of this species in Brazil (**Figure 1**). The tissue samples were extracted from euthanized specimens using anesthetic application to the skin (5% Lidocaine), according to recommendations of the Herpetological Animal Care and Use Committee (HACC) of the American Society of Ichthyologists and Herpetologists (available at: http://www.asih.org/publications) and approved by SISBIO/Institute Chico Mendes de Conservação da Biodiversidade as a condition for the concession license (number 14468-1/14468-4). Voucher specimens were deposited in the CFBH Amphibian Collection of the Paulista State University (UNESP) in Rio Claro, São Paulo, Brazil, and the Prof. Adão José Cardoso Museum of Zoology at Campinas State University (UNICAMP) in São Paulo, Brazil (see **Supporting information S1a**). We used *Pithecopus hypochondrialis* and *P. azureus*, two closely related species, plus *P. ayeaye*, *Phyllomedusa tarsius*, and *Callimedusa tomopterna*, as three successively distant (in this order) outgroup taxa (according to Duellman et al., 2016).

### Extraction, Amplification, and Sequencing of the DNA

The genomic DNA was extracted from liver and muscle tissue samples (preserved in ethanol 95%) using the TNES method (50 mM Tris, pH 7.5, 0.4 M NaCl, 20 mM EDTA, 0.5% SDS) (see Bruschi et al., 2012). We amplified and sequenced fragments of two mitochondrial genes, NADH dehydrogenase subunit 2 (*ND2*) and the ribosomal 16S gene (*16S*), and two nuclear loci, Rhodopsin exon 1 (*Rhod*) and Seven in Absentia Homolog 1 (*SiaH*) (primers used for each fragment are described in the Supporting information **Table S1B**). The polymerase chain reactions (PCRs) were run in a total volume of 25 µl containing 20–100 ng of the DNA template, 1× PCR buffer, 1.5 mM MgCl_2_, 7 pmol/µl of the forward and reverse primers, 1 mM of dNTPs, and 1 U of Taq polymerase. The amplicons were purified with exonuclease I (10 units) and SAP (1 unit) to remove unincorporated dNTPs and primers. Fragments were sequenced in an ABI PRISM^®^ 3100 Genetic Analyzer (Applied Biosystems, Foster City, CA, USA) in both directions, using the original amplification primers and BigDye terminator sequencing chemistry according to the manufacture’s protocol (Applied Biosystems, Foster City, CA, USA).

### Alignment and Phasing of the Nuclear Sequences

The sequences were inspected and edited in CodonCode Aligner 7.1.1. The *16S* and *ND2* mitochondrial sequences were aligned using the MUSCLE algorithm (Edgar, 2004) in MEGA 6.0 (Tamura et al., 2013); *SiaH* and *Rhod* nuclear sequences were aligned using the online version of the automatic alignment software MAFFT 7 (Katoh and Standley, 2013; available at: http://mafft.cbrc.jp/alignment/software), with the default options being applied. The phased haplotypes of the nuclear sequences were inferred by the Bayesian method implemented in PHASE 2.1 (Stephens and Donnelly, 2003), with the input files prepared with SEQPHASE (Flot, 2010; available online at: http://seqphase.mpg.de/seqphase/). We conducted the PHASE runs under default parameters (100 iterations with 100 burn-in and thinning interval of 10) and defined the minimum posterior probability of the haplotypes as 0.9.

### Haplotype Networks, Genetic Structure, and Diversity

Median-joining haplotype networks were constructed in POPART 1.7 (Bandelt et al., 1999; Leigh and Bryant, 2015). The species’ genetic structure was first assessed with a Bayesian analysis of population structure (BAPS) to determine the most probable number of clusters (*k*) in the dataset using BAPS 6.0 (Corander et al., 2008). We applied the “spatial clustering of individuals” option to the *16S*, *ND2*, *SiaH*, and *Rhod* datasets separately (as concatenating the fragments in mitochondrial versus nuclear datasets would reduce substantially the amount of information available for analyses, given most individuals had not all the four loci sequenced), allowing for a range of 1–30. The optimal *k* values were defined based on the highest marginal log-likelihood estimates.

We also investigated the population structure and estimated the intra- and interpopulation diversity indices in ARLEQUIN 3.5 (Excoffier and Lischer, 2010). We calculated the genetic distances and Φ_ST_ values between populations, considering each sampling location as a population. The correlation between the genetic and geographical distances was evaluated using the Mantel test performed in ARLEQUIN 3.5. We also estimated the genetic differentiation among BAPS groups (see Results) and ran analyses of molecular variance (AMOVA, Excoffier et al., 1992) to infer the hierarchical organization of this structure. The haplotype (h) and nucleotide (π) diversities and the number of polymorphic sites (S) were calculated for each location, BAPS group, and the total dataset.

### Phylogenetic Analyses and Molecular Dating

We inferred the phylogenetic relationships among lineages and dated the main divergence events using a Bayesian multilocus species tree approach (*BEAST; Heled and Drummond, 2010) in BEAST 1.8 (Drummond et al., 2012). We considered each BAPS group (northern, southern, and western groups, see *Results*) as a distinct lineage and pruned all loci datasets to one sequence per haplotype (i.e., excluding extra identical sequences) in each population. The trees were rooted with sequences from five outgroup species (*Pithecopus azureus*, *P. hypochondrialis*, *P. ayeaye*, *Phyllomedusa tarsius*, and *Callimedusa tomopterna*), and the nodes were calibrated using known *16S* and *ND2* substitution rates (0.28 and 0.957% per million years, respectively), as proposed by Lemmon et al. (2007) and Crawford (2003).

The best models of nucleotide substitution were selected *a priori* using the Akaike information criterion (AIC) in MEGA 6.0 (*16S*: GTR+G; *ND2*:TN93+G; *SiaH*: K2P+G+I and *Rhod*: HKY+G). We applied lognormal relaxed molecular clocks to the mitochondrial 16S and ND2 datasets but strict clocks to the *SiaH* and *Rho*d data, based on the striking low variability among these nuclear sequences (see Results) and the likelihood ratio tests also conducted in MEGA 6.0, which rejected the hypothesis of strict molecular clock only for *16S* and *ND2* loci. We estimated the mean substitution rates for the nuclear genes and selected the Yule process as the tree prior for three independent runs of 500 million generations, with samples being taken every 5,000 generations. We checked for the convergence of the runs and for high effective sample sizes (ESS > 200) in TRACER 1.6 (available at: http://tree.bio.ed.ac.uk/software/tracer/) and combined the trees from the independent runs in LOGCOMBINER 1.8. The first 50,000 trees (10%) of each run were discarded as burn-in, and the maximum clade credibility (MCC) of the species and independent gene trees were computed in TREEANNOTATOR 1.8. The trees were visualized in FIGTREE 1.4 (available at: http://tree.bio.ed.ac.uk/software/figtree/).

### Demographic Patterns

Signals of demographic events in the sampling locations, in the BAPS groups, and in the total dataset were investigated using neutrality tests [*D* (Tajima, 1989) and *Fs* (Fu, 1997) in ARLEQUIN 3.5, and *R2* (Ramos-Onsins and Rozas, 2000) in DNASP 1.5 (Librado and Rozas, 2009)]. We also verified the distribution of pairwise differences in sequences in each BAPS group through mismatch distribution histograms (Rogers and Harpending, 1992; Harpending, 1994), obtained in ARLEQUIN 3.5.

We also ran the extended Bayesian skyline plot (EBSP, Heled and Drummond, 2008) analyses in BEAST 1.8 to infer changes in effective population size over time in each BAPS group (except for the western group, for which only a small number of sequences was available and the variability was low, see Results). We unlinked substitution, clock, and tree models for all the loci and specified a linear model of population size. We used the same clock models and substitution rates applied to the *BEAST analysis, and the operators and initial values for the mean population sizes were adjusted to improve the MCMC mixing, as recommended in the tutorial on the BEAST website (available at: http://beast.bio.ed.ac.uk/). We applied two independent runs of 500 million generations for each group (with samples taken every 5,000 generations). We used TRACER 1.6 to check the convergence and quality of the parameters and LOGCOMBINER 1.8 to combine the resulting trees. The final plots were based on the output files produced for the combined results.

### Ecological Niche Modeling (ENMs)

We modeled the potential geographical distribution of *P. nordestinus* in present and past climate scenarios [120 ka, Last Interglacial Maximum (LIG); 21 ka, Last Glacial Maximum (LGM); and 6 ka–mid-Holocene (HOL)] based on ecological niche modeling proceedings (ENMs). For this, we used all 78 current known occurrence points for the species encompassing our field collected sites for genetic population structure and other points available in two databases: SpeciesLink (http://splink.cria.org.br/) and Global Biodiversity Information Facility—GBIF (www.gbif.org). After, we filtered the occurrence points to obtain only single-cell occurrences when compared with cell resolution to models building (4 × 4 km ∼16 km^2^ cell size, in Equador region) (**Table S2; Supporting information**).

We downloaded all 19 bioclimatic variables—of temporal scenario studied by us—that are available on the WorldClim Database (www.worldclim.org) and realized a factorial analysis according to Sobral-Souza et al. (2015) to select no-correlated variables using Caatinga/North Atlantic Forest biome delimitation as background. We used this background because it includes all occurrence points and encompass area for specie dispersion over time, two criteria for background selection proposed by Barve et al. (2011). Thus, we selected five variables, mean diurnal range (Bio 2), mean temperature of wettest quarter (Bio 8), mean temperature of driest quarter (Bio 9), annual precipitation (Bio 12), and precipitation of wettest mouth (Bio 13) (**Table S3; Supporting information**) to the model calibration. The models were built based on current climate condition and projected to mid-Holocene, LGM, and LIG climate using 2.5 arc-min resolution (4 × 4 km^2^). The LIG paleoclimate condition data were proposed by Otto-Bliesner et al. (2006) using only CCSM3 simulation, and for this, here, we chose to use only this simulation for all paleoclimate scenario and leave out other AOGCMs simulations.

Several algorithms are able to infer species distribution (Diniz-Filho et al., 2009). Here, we applied five different algorithms based on different modeling techniques such as distance, envelope, and machine learning (based on absence and pseudo-absence) to infer *Phitecopus nordestinus* distribution. We used 1) Bioclim (Nix, 1986), 2) Mahalanobis distance (Farber and Kadmon, 2003), 3) domain (Gower distance; Carpenter et al., 1993), 4) support vector machines (SVM) (Tax and Duin, 2004), and 5) maximum entropy (Phillips and Dudik, 2008). To test the built models we subset the occurrences points into training and testing folding, with 75% as 25% of occurrences, respectively. We repeated this procedure 20 times to decrease the spatial collinearity between folding. In addition, we calculated the maximum sensitivity and specificity threshold (mms) and the true skill statistics (TSS) value for each repetition. According to Allouche et al. (2006), acceptable models present TSS values over 0.5 to cut each map and transform them on binary map. We used the mms value as threshold because it is the better threshold to use when only presence (distance and envelope) techniques are used in ENMs proceedings (Liu et al., 2016). After this, we used the ensemble approach (Araújo and New, 2007) to calculate the frequency of prediction by grid cell to create a consensus map for each algorithm. Finally, we concatenated all consensus maps and then obtain a consensus map for each temporal climatic scenario (**Figure 1**).

### Model Testing With Approximate Bayesian Computation

**Figure 2:**
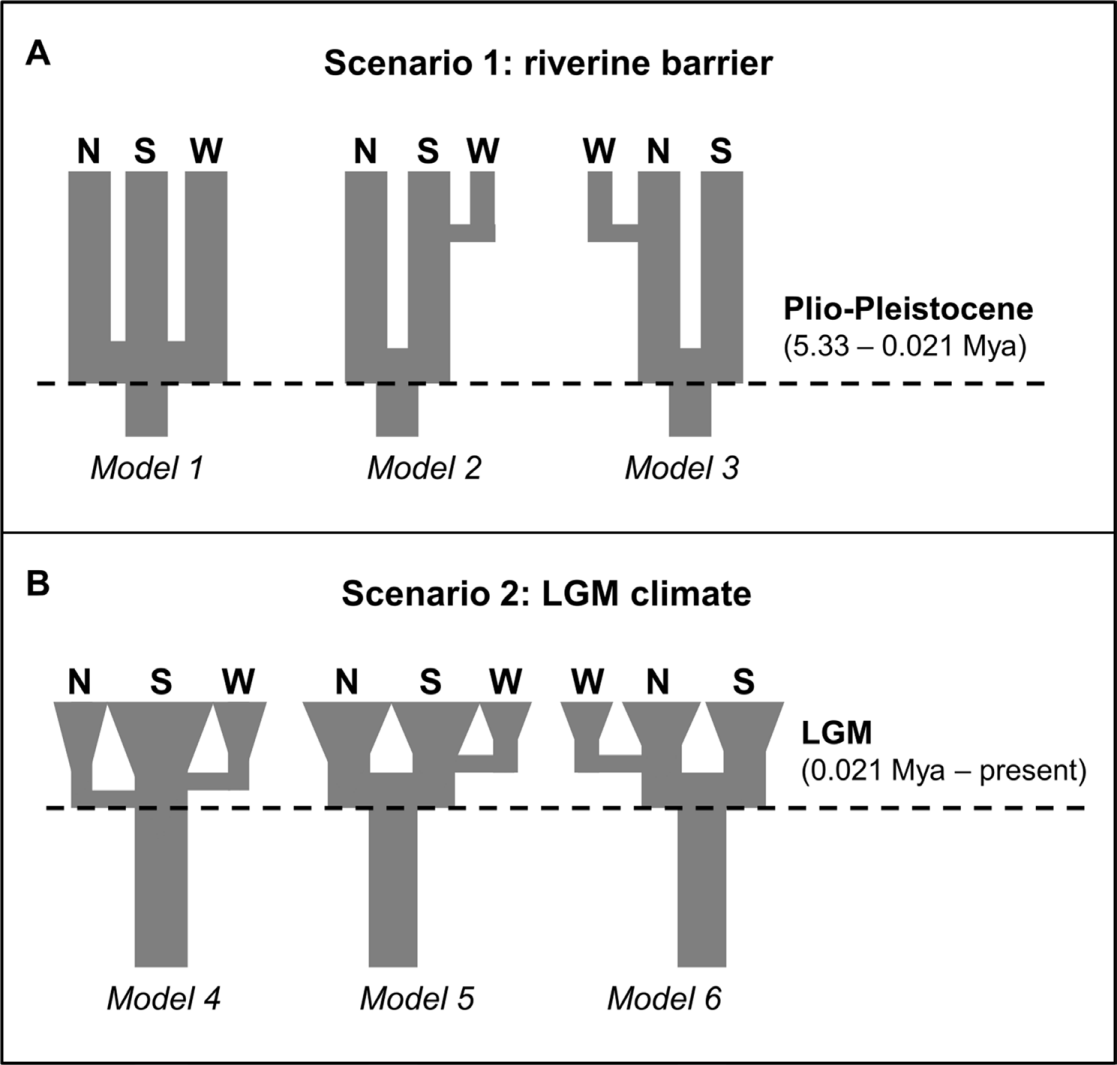
Alternative scenarios for diversification of the *P. nordestinus* populations of the South, North, and West lineages tested with multilocus approximate Bayesian computation (ABC). **(A)** Scenario 1 (models 1–3), the divergence between the Northern and Southern *P. nordestinus* populations is related to the relocation of the São Francisco River (SFR) during the Plio-Pleistocene and alternative three models. **(B)** Scenario 2 (models 4–6), the diversification observed in *P. nordestinus* is derived from the last glacial cycle as seen modeling in the ENM analysis (see **Figure 1**) (Pleistocene Refuges Hypothesis). See *Material and Methods* for more detail.

Based on our phylogenetic and ecological inferences, we designed two biogeographic scenarios to explain the geographical genetic structure detected in the preceding analyses to test the models in an approximate Bayesian computation (ABC) framework. In scenario 1 (models 1–3; **Figure 2A**), the divergence between the northern and southern *P. nordestinus* populations is related to the relocation of the São Francisco River during the Plio-Pleistocene, with three models: 1—simple vicariance among the northern, southern, and western populations; 2—divergence between northern and southern populations coinciding with the relocation of the São Francisco River, followed by the colonization of the western population by individuals from the southern population; and 3—divergence of the northern and southern populations by the São Francisco River, followed by the colonization of the western population by individuals from the northern population. In scenario 2 (models 4–6; **Figure 2B**), the diversification observed in *P. nordestinus* is derived from the last glacial cycle: 4—a single refugia during the LIG as seen in the ENM (**Figure 1E**) with posterior colonization of both northern and western populations; 5—two distinct refugia, corresponding to the northern and southern populations, followed by the colonization of the western population by individuals from the southern population; and 6—two distinct refugia, corresponding to the northern and southern populations, followed by the colonization of the western population by individuals from the northern population. In scenario 2, after the period of refugia during the LGM, we set populations to increase in number, represented by exponential growth. We first compared the models within scenarios and then compared the best model from each scenario to select the best overall hypothesis.

We developed custom R scripts to simulate data under each model using *ms* (Hudson, 2002). The simulations were conducted using the same loci length and sample sizes of the empirical data and set independently for each dataset, as concatenating mitochondrial versus nuclear sequences would considerably reduce the number of individuals available for analysis (since most of them had not all the regions sequenced); nevertheless, the summary statistics computed for all markers were considered jointly in the model choice. We estimated a unique prior interval for the effective population size (*N*_e_) and then used this parameter to delimit appropriate prior distributions for each *θ* (theta), considering *θ*_mit_ = *N*_e_ *μ* and *θ*_nuc_ = 4*N*_e_ *μ*. The mutation rates (per site per generation) for each region and the divergence times’ intervals were given by uniform distributions delimited by prior information obtained from *BEAST results. In scenario 1, the first divergence event encompasses the Plio-Pleistocene (5.33 to 0.12 Mya), while in scenario 2 it corresponds to the period from the LGM to the present time [values transformed in coalescent units (number of generations/4*N*_e_)]. The other parameters (number of individuals after a bottleneck event and growth rates) also followed uniform prior distributions (see more details in **Table S4; Supporting information**). The R scripts used are available on GitHub (https://github.com/luizbartoleti/Pnordestinus).

We first performed a round of 100,000 preliminary simulations per model with flat distributions of the parameters (**Table S4; Supporting information**). Based on these simulated data, we calculated summary statistics—total nucleotide diversity (π), number of segregating sites (ss), Tajima’s D (D), nucleotide diversity within (πw), and between (πb) populations—using a PERL script written by N. Takebayashi (available at http://raven.wrrb.uaf.edu/∼ntakebay/teaching/programming/coalsim/scripts/msSS.pl) and grouped them in vectors (2–5 statistics per vector) to find the combination that would optimize our model selection, i.e., which vector more accurately identifies the best model given the simulated data. This step was included because, while using few statistics could be less informative, including an excessive amount of values may provide not additional useful information for the estimation process and introduce stochastic noise. We performed a regression step with 10 simulations for each model and used them as PODs (pseudo-observed data). The best vector of summary statistics would enhance the probability of choosing the true model over the average probability of choosing any other model (Tsai and Carstens, 2013). We then conducted a parameter restriction to narrow their prior distributions and get a more reliable model choice. We used the “abc” package for R, version 1.4 (Csilléry et al., 2012) and applied a threshold of 1% of the simulations more similar to our empirical data based on a neural network rejection.

With the parameters restricted and the best vector of summary statistics (ss, D, and πb), we then performed more 300,000 simulations per model. Model selection was also made using the “abc” package and a threshold of 1% of the simulations. We calculated the posterior probabilities of the competing models to find the best model within each scenario and then compared these two models to find the best overall model. To investigate the robustness of our model choice, we performed a cross-validation analysis using 100 random simulated summary statistics as PODs to test if the models that we built were significantly different from each other (excluding the possibility of a random model selection). Finally, we evaluated the goodness-of-fit of the most probable model by comparing the statistics calculated from the retained simulations to our empirical data based on a principal component analysis (PCA), also with “abc” package in R.

## Results

### General Aspects of the Data

We obtained 151 sequences of *16S* gene (1035 base pairs or bps), 168 sequences of the *ND2* (813 bps), 155 sequences of *SiaH* (361 bps), and 189 of the *Rhod* locus (378 bps). All the reconstructed phased haplotypes had posterior probabilities higher than 0.9, which resulted in 310 phased *SiaH* sequences and 378 phased *Rhod* sequences.

### Phylogenetic Inferences, Haplotype Networks, Genetic Structure, and Diversity

**Figure 3:**
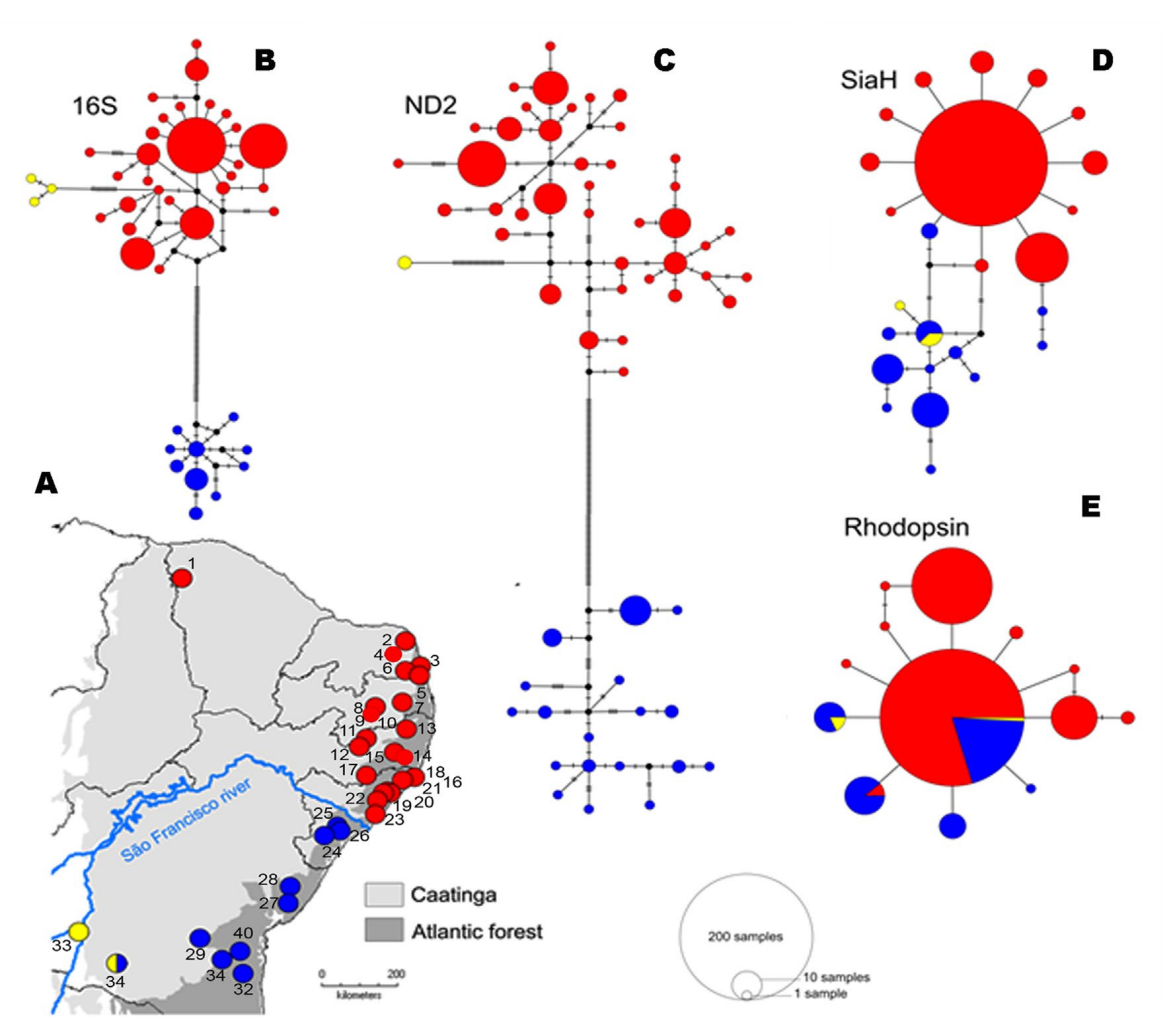
Spatial distribution **(A)** and haplotypes genealogies for Median-joining analysis of *16S* **(B)**, *ND2* **(C)**, *SiaH* **(D)**, and *Rhod* **(E)** fragment genes recovered of the *P. nordestinus* dataset. Each haplotype is represented by a circle whose area is proportional to its frequency (see legends). Samples are colored according to the haploclades recovered in the Bayesian topology and Bayesian analysis of population structure (BAPS) analysis. Red represent the Northern group, blue represent the Southern group, and yellow represent the West group. Note the blue and red lineages of the *P. nordestinus* bisected by current course of the SFR (light blue dashed). The numbers correspond to the localities named in BAPS analysis (**Supporting information—Figure S1**).

**Figure 4:**
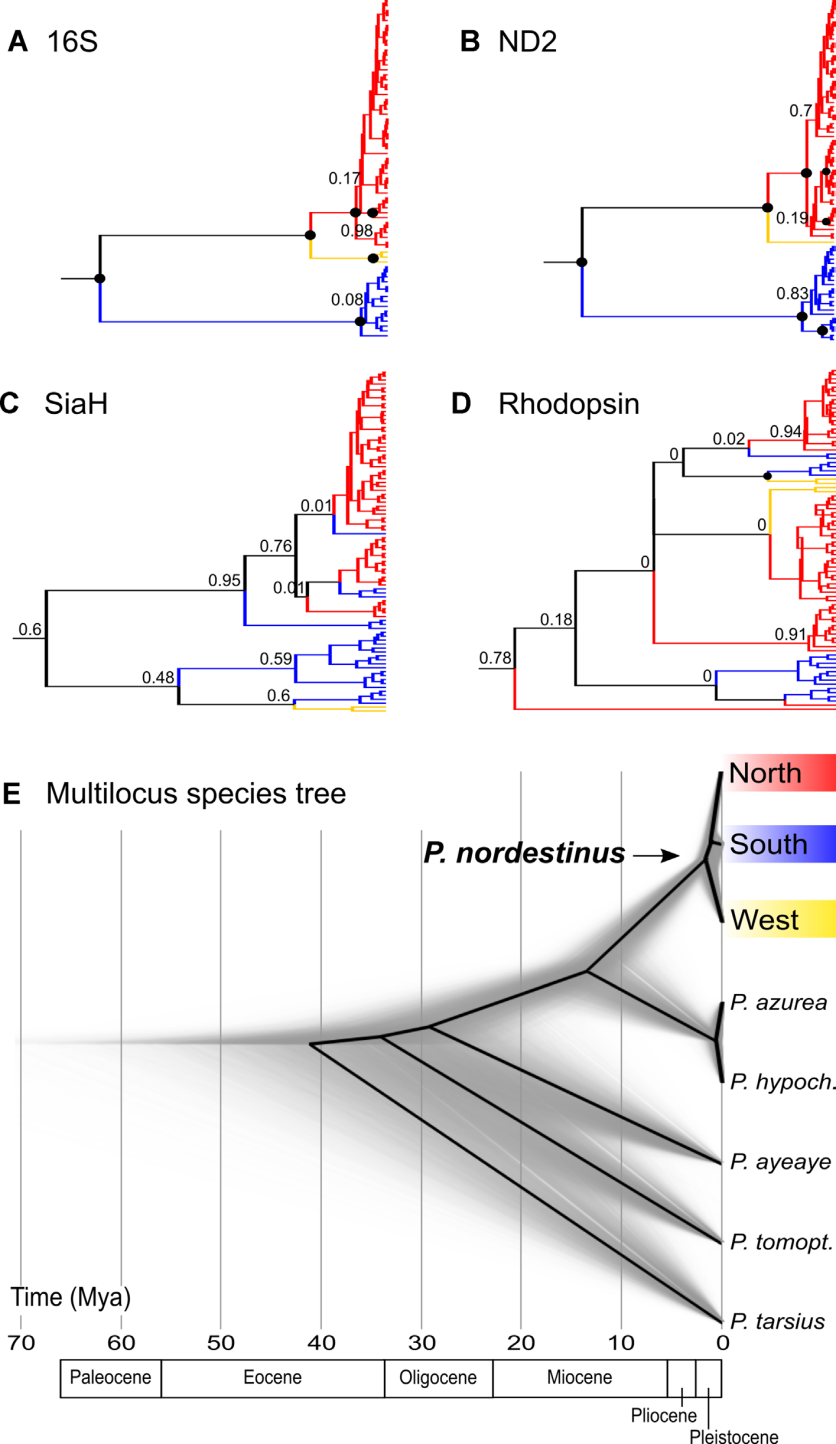
Multilocus Bayesian inferences (*BEAST) results showing the gene trees obtained for each marker **(A, B, C** and **D)** and the multilocus calibrated species tree **(E)**. In the gene trees, colors represent the mitochondrial groups pointed by BAPS (red = Northern group, blue = South group, and yellow = West group), and the black circles in the nodes indicate posterior probability values = 1.0.

The mitochondrial networks showed three major haplogroups separated by a large number of mutational steps, which correspond to distinct geographic regions (**Figures 3A–C**), but a less clear subdivision was found in the nuclear genes (**Figures 3D, E**). The lineage divergences based in the Bayesian phylogenetic inferences corroborate the haplotype networks and geographical structure (**Figure 4**). The mitochondrial gene (*16S* and *ND2*, **Figures 4A, B**, respectively) showed similar topologies and recovered the three major geographically structured *P. nordestinus* clades: the first includes specimens distributed on the north of SFR (Rio Grande do Norte, Ceará, Paraíba, Pernambuco, and Alagoas states, hereafter referred to Northern group); the second is composed by few haplotypes from western Bahia (Catieté and Aurelino Leal populations, hereafter referred to Western group); and a third clade genetically more distant from the other two that includes individuals collected in Sergipe and remaining localities in Bahia states, all distributed in South of the SFR (hereafter referred to Southern group). Nuclear gene trees, however, exhibited lower resolution in the internal nodes (**Figures 4C, D**), and two main mito-nuclear discordances in our topologies were detected: i) few specimens from Areia Branca and Laranjeiras, both located in Sergipe state (Southern group), were nested with Northern group (see *SIAH* tree—**Figure 3C**); and ii) Western group varied among nuclear markers, grouping with Northern group in *Rhod* tree and with Southern group in *SIAH* topology (**Figure 4**).

The BAPS results indicated an optimal partition of *k* = 3 for both *16S* and *ND2* datasets, as observed in the other analyses (see also **Figure S1** in supporting information). For the nuclear *SiaH* sequences, the analyses indicated an optimal subdivision of two groups (*k* = 2), mostly corresponding to the two margins of the SFR, and no partitioning (*k* = 1) in the case of the *Rhod* dataset (**Figure 1**, **Figure S1**). The only locality with samples assigned to different groups was Caetité, in western Bahia (**Supplemental Figure S1**), whereas in *16S* and *ND2* tree, it was nested with the northern group; in *SiaH* and *Rhod*, this sample was nested with the southern group.

The significant Φ_ST_ values between locations from different BAPS groups (excluding the estimates for Caetité vs. other populations since this location contains individuals grouped in different genetic clusters) were consistently higher and varied from 0.94 to 1 (for *16S*), 0.42 to 1 (*ND2*), 0.42 to 1 (*SiaH*), and 0.12 to 0.44 (*Rhod*) (**Support information—Tables S4–S7**). Most pairwise Φ_ST_ values between populations grouped in the same BAPS cluster were nonsignificant, being the significant estimates usually higher among northern populations and varying from 0.12 to 1 (*16S*), 0.08 to 1 (*ND2*), 0.03 to 0.54 (SiaH), and 0.08 to 0.37 (*Rhod*) (**Tables S5–S8**). The genetic distances were also generally smaller between locations of the same group for all loci (**Support information’s—Tables S9–S12**). The Mantel tests indicated significant correlations between geographical and Φ_ST_/genetic distances when the total datasets were analyzed, but no evidence of isolation by distance was detected when each BAPS group was analyzed separately (data not shown).

The AMOVA indicated that most of the genetic variance arises among the Northern, Southern, and Western groups for the *16S* (94.9%), *ND2* (92.9%), and *SiaH* (81.9%) datasets but not for the *Rhod* sequences (17.7%, **Table 1**). The pairwise Φ_ST_ values were also high for all markers except *Rhod* (**Table 2**), and the corrected genetic distances varied from 12.3 to 61.6 pairwise differences for the *16*S sequences, 23.9 to 87.0 for *ND2*, 0.8 to 4.0 for *SiaH*, and 0.1 to 0.2 for *Rhod* (**Table 3**).

**Table 1:** Analysis of molecular variance (AMOVA) for the mitochondrial and nuclear dataset of the *P. nordestinus* populations. The hierarchical tests were designed considering BAPS groups.

Region	Source of variation	Variation percentage
***16S***	Among groups	94.89
	Within groups	5.11
***ND2***	Among groups	92.95
	Within groups	7.05
***SiaH***	Among groups	81.89
	Within groups	18.11
***Rhod***	Among groups	17.71
	Within groups	82.29

**Table 2:** Pairwise Φ_ST_ values between north, south, and west groups recovered in BAPS analysis of the *P. nordestinus* populations. In bold, the statistical significant results.

Region		North	South
***16S***	South	0.954	
	West	0.809	0.948
***ND2***	South	0.932	
	West	0.808	0.905
***SiaH***	South	0.818	
	West	0.899	0.166
***Rhod***	South	0.172	
	West	0.289	0.130

**Table 3:** Genetic distances between north, south, and west groups of the *P. nordestinus*.

Region		North	South	West
***16S***	North	2.885	64.617	14.457
	South	61.379	3.591	64.035
	West	12.348	61.573	1.333
***ND2***	North	5.410	91.573	26.618
	South	84.293	9.151	91.543
	West	23.914	86.967	0.000
***SiaH***	North	0.448	5.032	2.337
	South	3.476	2.663	2.337
	West	3.999	0.756	0.500
***Rhod***	North	0.603	0.799	0.873
	South	0.130	0.733	0.817
	West	0.238	0.117	0.667

Above the diagonals, the average number of sequences’ pairwise differences between groups (D); below the diagonals, the corrected average number of pairwise differences (DA). In gray, the average number of differences within each group.

The total and within-group haplotype and nucleotide diversities were higher for the mitochondrial datasets (**Table 4**). The variability of the southern group was slightly higher for all markers, and the pattern within sites varied considerably, due in part to the reduced sample sizes (**Table 4**, **Support information—Table S13**).

**Table 4:** Diversity indices and neutrality tests’ result for each BAPS group and for the total datasets of *P. nordestinus*.

	N	h	S	Hd (s.d).	π (s.d).	*D*	*Fs*	*R2*
*16S*								
North	129	31	37	0.86 (0.02)	0.003 (0.002)	−1.80 (*)	−18.50 (**)	0.04 (*)
South	19	10	21	0.88 (0.06)	0.003 (0.002)	−1.46	−2.24	0.08 (**)
West	3	3	2	1 (0.27)	0.001 (0.001)	0.00	−1.22	0.24
Total	151	44	107	0.89 (0.02)	0.017 (0.008)	−0.43	−1.42	0.07
*ND2*								
North	131	40	54	0.93 (0.01)	**0.007 (0.003)**	−1.41 (*)	−17.59 (**)	0.05 (*)
South	35	21	44	0.9 (0.04)	0.011 (0.006)	−0.52	−4.06	0.09
West	2	1	0	0 (0)	0 (0)	0.00	0.00	0.00
Total	168	62	167	0.95 (0.01)	0.042 (0.021)	0.57	0.00	0.10
*SiaH*								
North	260	12	11	0.41 (0.04)	0.001 (0.001)	−1.74 (**)	−10.66 (**)	0.02
South	46	14	9	0.83 (0.04)	0.007 (0.004)	0.85	−4.23	0.15
West	4	2	1	0.5 (0.26)	0.001 (0.002)	−0.61	0.17	0.43
Total	310	25	19	0.57 (0.03)	0.005 (0.003)	−1.08	−13.47 (**)	0.04
*Rhod*								
North	292	10	7	0.53 (0.03)	0.002 (0.002)	−0.94	−5.28	0.04
South	82	5	4	0.61 (0.05)	0.003 (0.002)	−0.18	−0.55	0.09
West	4	2	1	0.67 (0.2)	0.002 (0.003)	1.63	0.54	0.33
Total	378	13	10	0.57 (0.03)	0.002 (0.002)	−1.21	−7.90 (**)	0.03

N, number of sequences; h, number of haplotypes; S, number of segregating sites; Hd, haplotype diversity; π, nucleotide diversity; s.d., standard deviation; D, Tajima’s D; Fs, Fu’s Fs; R2, Rozas and Ramos-Onsis’ R2. In bold, the statistical significant values for the neutrality tests (*p < 0.05, **p < 0.02).

### Divergence Time Estimation

The *BEAST analysis indicated that *P. nordestinus* diverged from the outgroup at ca. 13.24 Mya (95% HPD = 8–19 Mya: **Figure 4E**). The intraspecific lineages’ divergences started at ca. 1.57 Mya (95% HPD = 0.84–2.61 Mya); however, the phylogenetic relationships among the three lineages were not clearly resolved, given that the divergence time’ intervals overlap, and the posterior probability of the north-south clade is low (PP = 0.6, **Figure 4E**).

The trees for the mitochondrial genes presented highly congruent topologies, indicating that the southern group lineage split off first [16S: 9.99 Mya (95% HPD = 6.1–14.16); ND2: 8.75 Mya (95% HPD = 5.23–12.78)], while the northern and western samples were more closely related and diverged only more recently [*16S*: 2.68 Mya (95% HPD = 1.58–4.03); *ND2*: 2.3 Mya (95% HPD = 1.38–3.73), **Figure 4**; **Supporting information Figure 2**]. The *SiaH* gene tree presented a less marked differentiation among the three lineages, and, in contrast with the mitochondrial trees, the western samples were more closely related to the southern group; however, most of the nodes have low statistical support (**Figure 4C**). The *Rhod* tree exhibited almost no structuring of the groups and nodes’ posterior probabilities even lower (**Figure 4D**; **Supporting information**).

### Demographic Patterns

**Figure 5:**
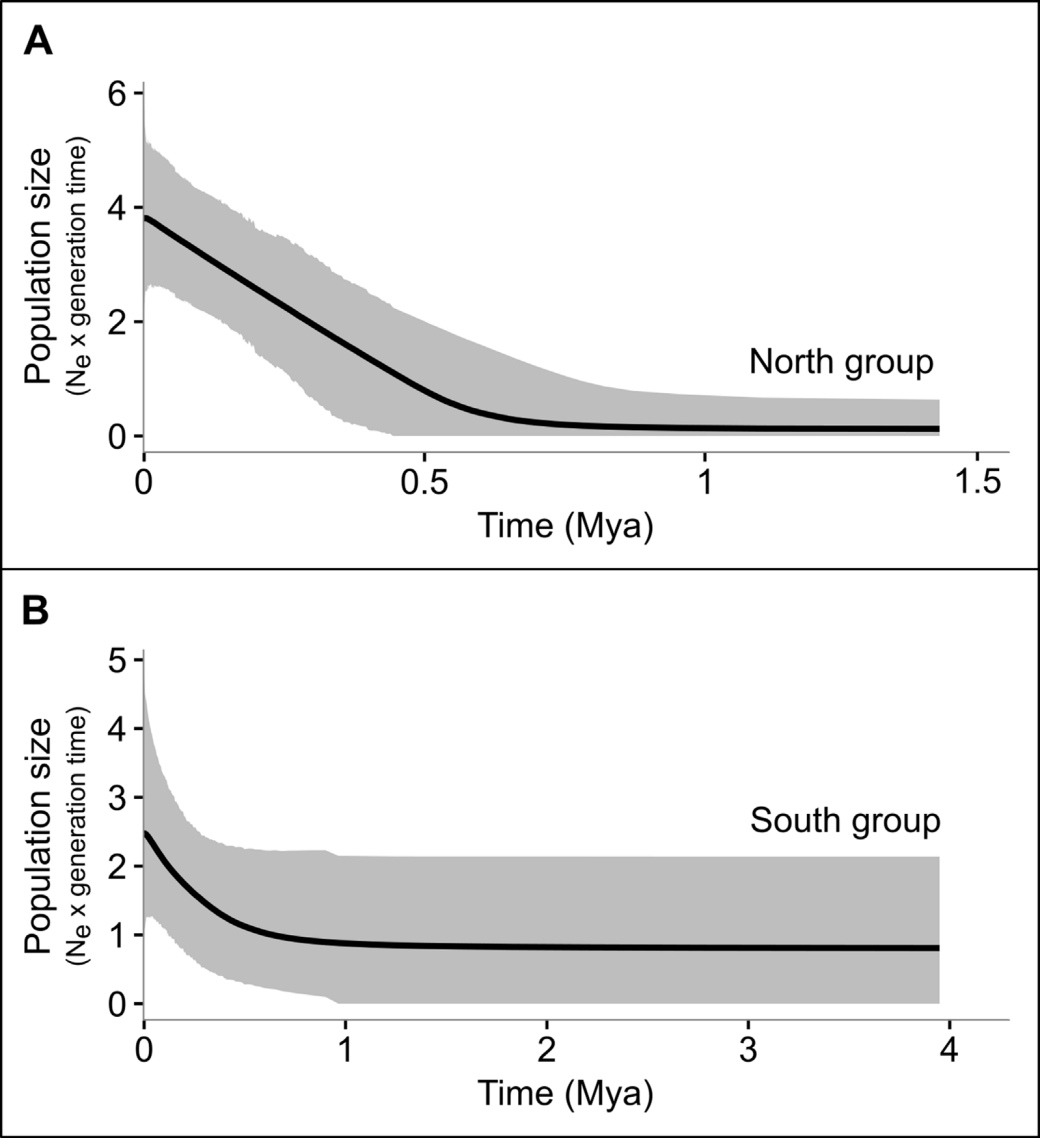
Multilocus extended Bayesian skyline plots (EBSP) illustrating effective population sizes (Ne × generation) though time (in Mya) of the north **(A)** and South **(B)** groups of *P. nordestinus*. Gray shades represent the 95% higher posterior density intervals.

All the neutrality tests revealed signs of demographic expansion (evidenced by the negative value in *D* and *Fs* tests, and low values in *R2* test) in the northern group, based on the mitochondrial markers, a pattern also detected in the *D* and *Fs* tests for *SiaH* sequences (**Table 4**). In the southern group, only *R2* test on the *16S* sequences indicated population expansion, and none of the results were significant for the western group, probably due to its smaller sample size (**Table 4**). Few significant results were found within geographical locations, except for some northern populations (despite the fact that they were not totally congruent among markers and tests **Table S14**). For the complete datasets, only the *SiaH* and *Rhod* sequences presented significant *Fs* values (**Table 4**). The EBSP analyses indicated population growth in both northern and western groups, starting at ca. 0.5 Mya (**Figure 5**).

### Ecological Niche Models

The ENMs suggested a robust geographical expansion during LIG to current climate conditions (**Figure 1**). During LIG, the only high suitability area was located in southern region of São Francisco River (**Figure 1E**), suggesting an influence of São Francisco Riverine Basin on species distribution. Between LIG to LGM climate conditions, a marked geographical expansion occurred in the southern–northern direction with expansion to western region (**Figure 1D, E**). After LGM, the geographical distribution has remained stable (**Figures 1B–D**).

### Model Testing With Approximate Bayesian Computation

The best supported model was model 2 (**Table 5**): an ancient split between the northern and southern populations resulting from the relocation of the SFR, with posterior founding of the western region by individuals from the southern populations. This analysis had a high Bayes factor (BF > 400), which strongly supports the conclusion that the diversification of the population was due to the relocation of the river (**Table 5**).

**Table 5:** Results of the ABC model selection assuming two major scenarios for diversification in *P. nordestinus* lineages (Riverine barrier versus Climate refugia—Figure 2).

Scenario	mODEL	Within scenarios	Among Scenarios	BAYES FACTOR
**1** **Riverine barrier**	1	0.2729	–	
	2	**0.6487**	**0.9976**	411.0854
	3	0.0785	–	
**2** **Climate refugia**	4	0.1782	–	
	5	**0.6136**	0.0024	
	6	0.2082	–	

The best model in each scenario (the one with the highest posterior probability—in bold) were used to perform the analysis among scenarios, which ultimately selected the best-fitting model for our data.

The confusion matrix generated by the cross-validation (**Supplemental Figure S3**, **Table S15**) indicated that the PODs matched satisfactorily the models under which they were generated, and the correct models were always more frequently recovered (except for model 5 evaluation, which indicated model 6 as the correct model with a slightly higher frequency). The analysis showed a clear discrimination between simulations from different scenarios; the higher number of erroneously recovered models in the same scenario, though, indicates that within-scenario demographic inferences should be taken cautiously and further explored in following studies. The goodness-of-fit PCA analysis confirmed that the summary statistics simulated under model 2 are considerably similar to our empirical values (**Figure S4, Supporting information**).

## Discussion

### Strong Phylogeographic Break in *P. nordestinus*

The present study included a broader sample of populations from the geographic range of *P. nordestinus*, permitting a better resolution of the sources of the genetic variation reported by Faivovich et al. (2010). Our results indicated that *P. nordestinus* is composed of distinct geographically structured lineages, which probably diverged due to geological processes involved in the translocation of São Francisco river.

The major phylogeographical break was detected between the populations located at the north versus south of SFR (**Figures 3**, **4**, **Tables 1**–**3** and **5**). The divergence between these lineages was profound [ca. 1.5 Mya (0.8–2.6 Mya); see Results] and consistent with the presence of such topographic barrier, dated to Early-to-Mid Cretaceous (Potter, 1997). As expected under the Riverine Barrier hypothesis’ predictions, this barrier would reduce gene flow between populations in opposite riverbanks over time, resulting in greater genetic differentiation in comparison with that found among populations on the same margin (Rull, 2011). Our ABC model testing also recovered strong probabilistic support for this scenario, confirming the riverine barrier hypothesis as the most plausible vicariance event for *P. nordestinus*. The SFR has long been considered an important geographic barrier in the Atlantic Forest biome, which has influenced vicariant processes at both intergeneric (Rodrigues, 1996; Rodrigues and Juncá, 2006; Siedchlag et al., 2010) and intraspecific levels (Pelegrino et al., 2005; Passoni et al., 2008; Siedchlag et al., 2010; Nascimento et al., 2011; Werneck et al., 2012; Nascimento et al., 2013). The diversification time estimates to lineages of the rodents (Nascimento et al., 2013) and dipterans (Coutinho-Abreu et al., 2008), lizards (Werneck et al., 2015) from opposite SFR banks during Plio-Pleistocene transition, time frame coincident with North and South split detected in *P. nordestinus*.

During the Plio-Pleistocene transition, the uplifting of the Parnaíba River basin resulted in a shift in São Francisco River drainage, which followed a transversal geological fault on the northeastern coast of Brazil (King, 1956; Mabesoone, 1994). The split of the northern and southern groups of *P. nordestinus* was also dated to this period; this geomorphological event had a profound effect on the local biodiversity: examples of allopatric speciation and lineages’ differentiation in this region include lizards of the genera *Eurolophosaurus* (Passoni et al., 2008), *Calyptommatus* (Schidchlag et al., 2011) and *Phyllopezus* (Werneck et al., 2012), and the Caatinga species complex *Tropidurus semeitaeniatus* (Werneck et al., 2015); rodents of the genus *Calomys* (Nascimento et al., 2011); and dipterans of the genus *Lutzomyia* (Coutinho-Abreu et al., 2008).

While landscape changes, such as shifts in the drainage of major rivers, have played a clear role in the differentiation of lineages, subsequent during this historical process, intermediate paleo-drainage phases would have occurred, as indicated by debris flows and fluvial deposits dated to the Pleistocene (Lima et al., 2014). These events promoting micro-climatic alterations that demanded differential ecological responses in different taxa. This reinforces the role of the continuous landscape changes during the Pleistocene in the region of São Francisco river (Lima et al., 2014) in shaping the current genetic diversity and structure of the populations. Thus, both ancient geographic barriers and Pleistocene climate changes could have contributed to the patterns observed, and discriminating which event was more relevant for *P. nordestinus* history is not a simple task. Given their overlapping time frame, the genomic signatures of these two processes might be confounded or even cancelled out (Mona et al., 2014; Thomé et al., 2014). In addition, the dispersal abilities and generation time of each species should be considered to evaluate the role of the climatic changes as source of the molecular diversity of species, as validated by Arenas et al. (2014).

We also recovered two areas of predicted stability (refugia) during the period of the LGM, as pointed by our niche models (**Figure 1**). These areas are separated by the São Francisco River and coincide with the Pernambuco and Bahia centers of endemism proposed by Carnaval and Moritz (2008), which presumably promoted the divergence patterns in several organisms (Carnaval et al., 2009; Menezes et al., 2016). However, our molecular dating indicated that the divergence between northern versus southern *P. nordestinus* groups occurred in the Plio-Pleistocene period; thus, our results suggest that the Pleistocene refugia would have reinforced rather than originate the geographic patterns found in our dataset. We should also consider that we inferred the effects of climate condition based on paleo-simulation of the last 120 kya (LIG), but distinct proxies indicate that the multiple cycles of Quaternary glaciations have presented equivalent climatic oscillations over at least the last 800 ka (see Lisieck and Raymo, 2005). Thus, the climate changes that occurred in Plio-Pleistocene, when São Francisco River Basin was modified, can also support the genetic pattern showed here. Therefore, SFR course modification and climate change associated to Plio/Pleistocene period (5 to 1.5 Ma) may, together, explain *P. nordestinus* diversification better than the hypothesis of LGM climate change.

Ours results also detected signals of demographic expansion for both the northern and southern groups of *P. nordestinus*, which partially violates the predictions of the riverine barrier hypothesis (Moritz et al., 2000). However, the EBP analyses showed that population growth in both lineages started at ca. 0.5 Mya, which suggest that Pleistocene climatic shifts possibly have induced such demographic changes after the lineages split. Demographic oscillations are expected in forest-dwelling species affected by Pleistocene climatic shifts in the Atlantic Forest (Carnaval et al., 2009; Batalha-Filho et al., 2012; Dantas et al., 2014), which would be reflected in low genetic diversity, and a strong signal of recent population expansion. This signature was observed mainly in *P. nordestinus* northern populations, as we detected significant negative results of *Tajima’s D*, *Fu* and *Li’s* statistic, and *R2* tests. According to EMN predictions, *P. nordestinus* northern group may have been isolated within the Pernambuco refugia (Carnaval and Moritz, 2008) during the LGM, which left a footprint in the genome of these populations. In the southern group, by contrast, our demographic tests detected a major signal of expansion only in the *16S* marker (*R2* test). Although the Bahia refugia (Carnaval and Moritz, 2008), the largest center of endemism of the Atlantic Forest, is located in the central portion of the biome, we sampled only few sites in this region; thus, the weaker signs of historical demographic processes in these populations might be a result of our lower sampling.

### Incongruence Between Markers and Secondary Contact Among Evolutionary Lineages

We obtained few conflicting results for the mitochondrial and nuclear markers (gene trees, BAPS, and network genealogy) with respect to specimens from Areia Branca and Laranjeiras (hereafter referred to an ABL group), both located in the Sergipe state, which are located on the southern margin of the São Francisco River. These samples were grouped with the northern clade in the *SiaH* and *Rhod* fragment analyses, whereas mitochondrial markers (*16S* and *ND2*) was nested in the southern clade. This incongruence is expected in recently diverged lineages (and often causes noise in biogeographic studies; see review of the Toews and Brelsford, 2012) and may be a result of incomplete lineage sorting or mitochondrial introgression resulting from secondary contact between evolutionary lineages. In fact, the longer coalescence time of the nuclear markers in comparison with those of the mitochondrial genome implies that lineage sorting is more easily detected in the mitochondrial markers. While this can be ruled out in some cases, if the sorting of lineages is the source of the disagreement between the mitochondrial and nuclear signals, we would not expect any biogeographic pattern in the nuclear topology to affect populations that present well-defined structure in the mitochondrial trees (Funk and Omland, 2003). In the case of the ABL group, in fact, the mitochondrial sequences were highly divergent in comparison with the northern margin, even though these localities grouped with the northern clade in the analysis of the *SIAH* sequence. In this case, incomplete lineage sorting may be related to the formation of intermediate paleo-drainage basins during the Pleistocene, as indicated by the presence of debris flows and fluvial deposits (Lima et al., 2014) dated to ca. 450 ka, indicating that the course of the lower São Francisco River ran to the south of its present position. In this historical configuration, Areia Branca and Laranjeiras, which are located south of the current SFR curse, would have grouped with the northern populations, and this was confirmed by the nuclear markers, reflecting lower mutation rates, recombination effects, and higher effective population size in comparison with the mitochondrial genome, which may represent a more recent separation. A similar pattern of landscape restructuring may account for the sharing of haplotypes between lizard populations of the same region that are currently isolated by the São Francisco River (Werneck et al., 2015).

Alternatively, sex-biased dispersal may explain the incongruence detected between mitochondrial and nuclear markers in the regional scale. When individuals of one sex showed dispersal or phylopatric behavior, the signature of the genetic differentiation between populations could be recovered when estimate using uni- and biparental markers (Prugnolle and Meeus, 2002; Liebgold et al., 2011). Unfortunately, due to the few female samples, we cannot verify the sex-biased behavior signal as the source of this incongruence. To validate these hypotheses to require combine observational data of the mating system and adequate female sampling, future studies specifically designed will be beneficial to explain this incongruence.

### Incomplete Lineage Sorting or Secondary Contact Among Evolutionary Lineages?

The evolutionary history of the Western *P. nordestinus* populations requires more detailed analyses. While the mitochondrial markers indicated a link with the northern group, the nuclear markers grouped these sequences with either the southern (*SiaH*) or northern (*Rhod*) groups. Our ABC framework suggested that the western region was recently (post-LGM) colonized by individuals from the southern population after the main northern–southern split. This prediction is consistent with the signal of population expansion found in the southern group (*16S* fragment), even though no evidence of demographic change was found in the western clade (possibly due to the small sample size). Expansion after the vicariant event would coincide with the warmer climate following the glaciations of the Pleistocene and may account for the colonization of the western area.

*P. nordestinus* is a lowland species distributed throughout much of northeastern Brazil in areas of Caatinga or associated habitats of the Atlantic Forest and Cerrado morphoclimatic domain (Caramaschi, 2006; Faivovich et al., 2010). This species is normally found near water bodies in open areas with predominantly herbaceous vegetation. Caldas et al. (2014) reported that populations in Caatinga and Atlantic Forest habitats occupy similar microhabitats and use similar spatial resources, which may reflect a degree of niche conservation, even though body sizes vary between these biomes. The historical transition of ancient populations between these regions would thus be a plausible explanation for the colonization of the western populations. In this case, we have interpreted the disagreement between the mitochondrial and nuclear datasets as the retention of ancestral polymorphism through incomplete lineage sorting. Nevertheless, we agree that the short divergence time between the southern and western populations and the sharing of a number of haplotypes with the northern populations may require the testing of a more comprehensive hypothesis to account for these disagreements. Even so, possible gene flow between southern and northern populations within a putative secondary contact zone might also have created cytonuclear noise in the phylogenetic inferences due to introgression (Toews and Brelsford, 2012). However, if the signal detected in our topologies resulted from secondary contact, we would also expect to find demographic expansion in the southern group creating a hybridization zone in the region of the middle São Francisco River, with a more heterogeneous signal of haplotype and nucleotide diversity and a greater number of haplotypes shared between the southern and northern populations (Sequeira et al., 2011), although we found weak evidence of this in our data. This hypothesis cannot be discarded altogether, given the phylogeographical patterns in the genetic structure of lizards (Werneck et al., 2015) and rodents (Nascimento et al., 2013), which have demonstrated the potential existence of the past connections between the opposite margins of the São Francisco River, coinciding with the region occupied by the western clade of *P. nordestinus*.

These connections are validated by the paleological evidence of the increasingly hot and dry climate in the region following the LGM, which led to a reduction in the volume of water in the middle São Francisco River. These conditions also favored the expansion of the Caatinga and would have resulted in contact between opposite margins of the São Francisco in western Bahia. The islands found in the river at Xique-Xique are considered to be a relic of this period of Pleistocene climatic change (Barreto, 1996; Nascimento et al., 2013), and additional sampling of *P. nordestinus* in this region may provide important insights into the existence of a possible secondary contact zone between the northern and southern lineages.

## Conclusion

Our data support the riverine barrier model as the origin of the genetic structure detected among *P. nordestinus* populations, in particular the historical shifts in the course of the São Francisco River. This river has acted as a physical barrier to gene flow between populations from opposite margins, resulting in the lineage divergence observed. In addition, we found evidence that genetic differences were reinforced by Pleistocene climate oscillations, which includes a genomic signature of post-LGM demographic population expansion. Despite the fact that phylogenetic systematics were beyond the scope of this paper, we can only emphasize the need for a complete taxonomic review of the species, including the definition of species ranges and morphological diagnoses, once that our data revealed two units that have evolved independently. The recognition of these two independent lineages also has important implications for their conservation in northeastern Brazil as well as for the understanding of ecological and evolutionary patterns, in particular the patterns of species diversification in the Atlantic Forest.

## Ethics Statement

The examined specimens were collected with appropriate permission under authorization number 14468-1/14468-4 issued by SISBIO/Instituto Chico Mendes de Conservação da Biodiversidade. For the subsequent techniques, all tissue sampling were extracted from euthanized specimens using anesthetic application over the skin (5% Lidocaine) to minimize animal suffering, according to recommendations of the Herpetological Animal Care and Use Committee (HACC) of the American Society of Ichthyologists and Herpetologists (available at: http://www.asih.org/publications), and approved by SISBIO/Institute Chico Mendes de Conservação da Biodiversidade during the process of the concession license.

## Author Contributions

DB: designed and coordinated the study of molecular data, provided molecular dataset, analysed the data, and draft the manuscript. EP: analysed the data, discuss results and helped draft the manuscript. LL: discuss results and helped draft the manuscript. LB: ABC analysis and helped draft the manuscript. TS-S: ENM analysis and helped draft the manuscript. SMRP: designed and coordinated the study of molecular data, discuss results and helped draft results. All authors have read and approved the final version submitted.

## Conflict of Interest Statement

The authors declare that the research was conducted in the absence of any commercial or financial relationships that could be construed as a potential conflict of interest.

## Supplementary Material

Figure S1 Bayesian Analyses of Population Structure (BAPS) results. The colors represent the subdivisions indicated, and the mitochondrial results are shown in Figure 3.

Figure S2 Mitochondrial and nuclear tree obtained in *BEAST analysis. (**A**) 16S gene; (**B**) ND2; (**C**) SiaH; (**D**) Rhod.

Figure S3 Confusion matrix generated by the cross-validation performed using the “abc” package on R.

Figure S4 Goodness-of-fit PCA analysis of the summary statistics simulated under Model 2.
