# Supplementary figures and images for: Signature of the Paleo-Course Changes in the São Francisco River as Source of Genetic Structure in Neotropical Pithecopus nordestinus (Phyllomedusinae, Anura) Treefrog
Source: Front Genet. 2019 Aug 14;10:728. doi: 10.3389/fgene.2019.00728 (PMC6702341; doi:10.3389/fgene.2019.00728)

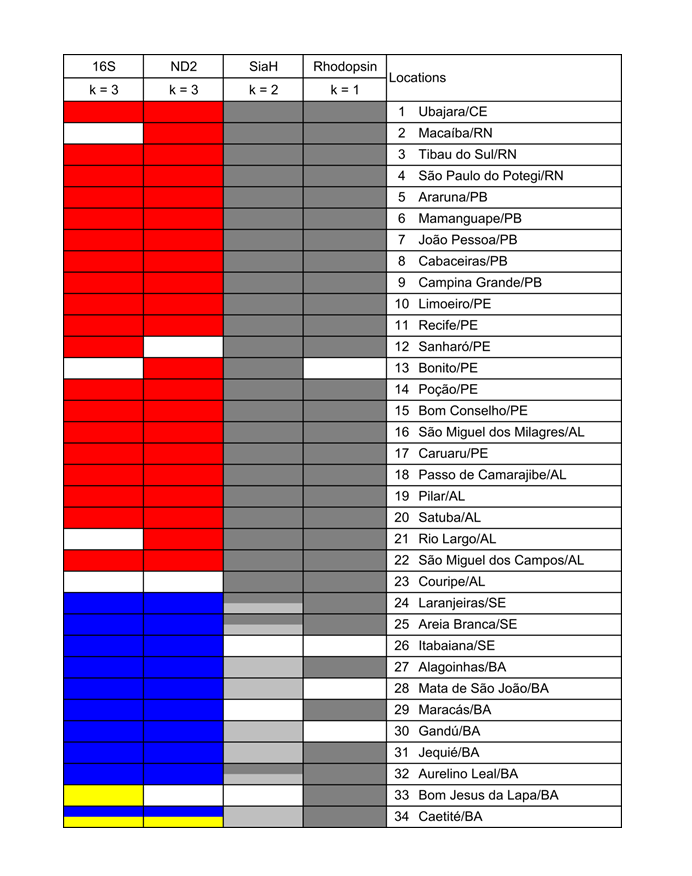

Supplement: Figure S1 — Bayesian Analyses of Population Structure (BAPS) results. The colors represent the subdivisions indicated, and the mitochondrial results are shown in Figure 3. [file Image_1.tif]

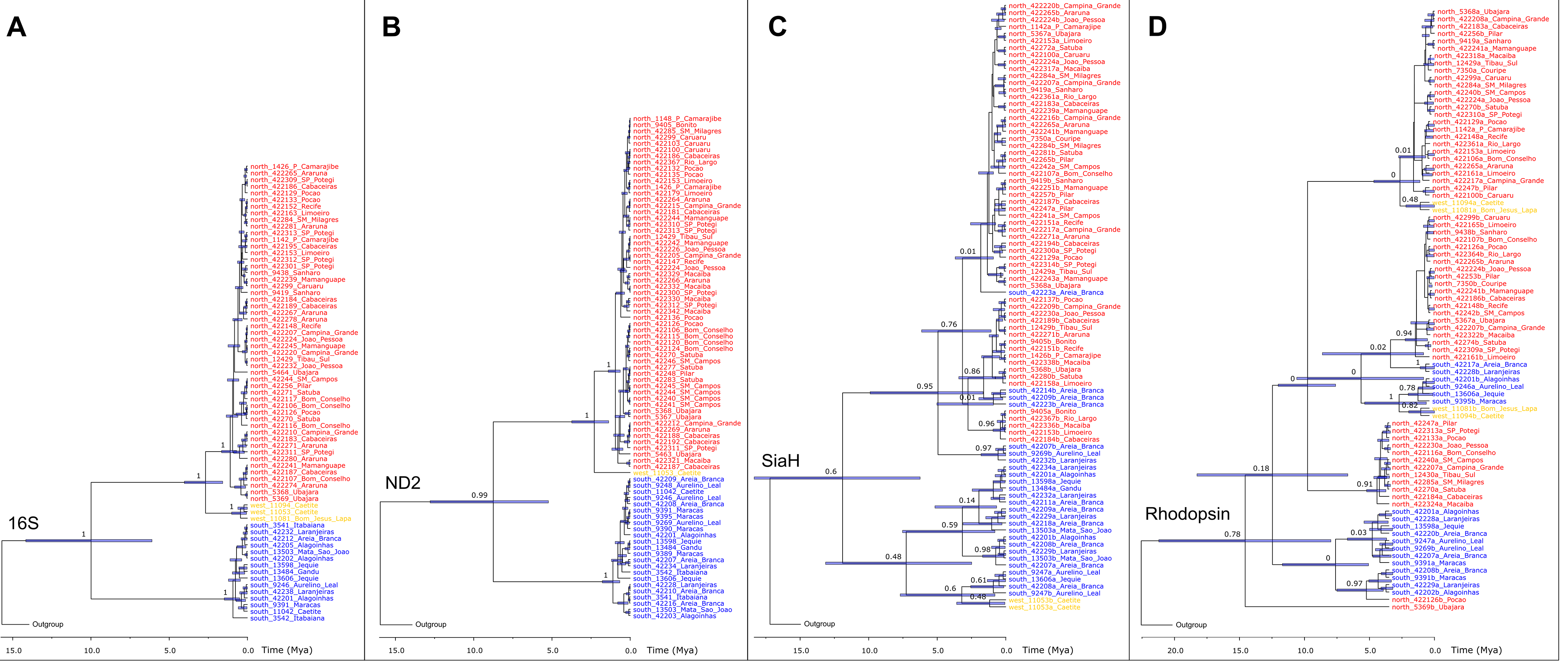

Supplement: Figure S2 — Mitochondrial and nuclear tree obtained in *BEAST analysis. (A) 16S gene; (B) ND2; (C) SiaH; (D) Rhod. [file Image_2.tif]

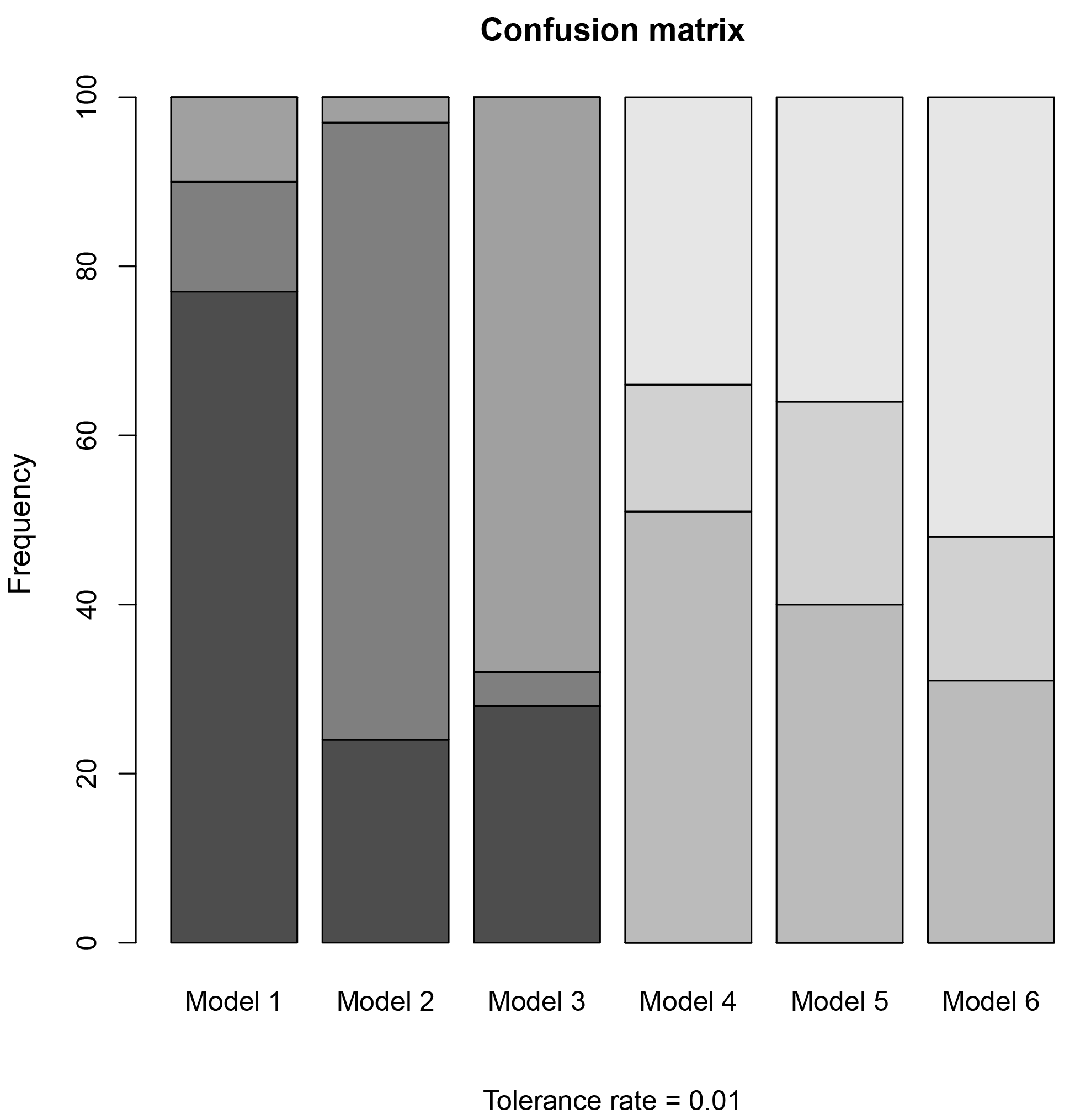

Supplement: Figure S3 — Confusion matrix generated by the cross-validation performed using the “abc” package on R. [file Image_3.tif]

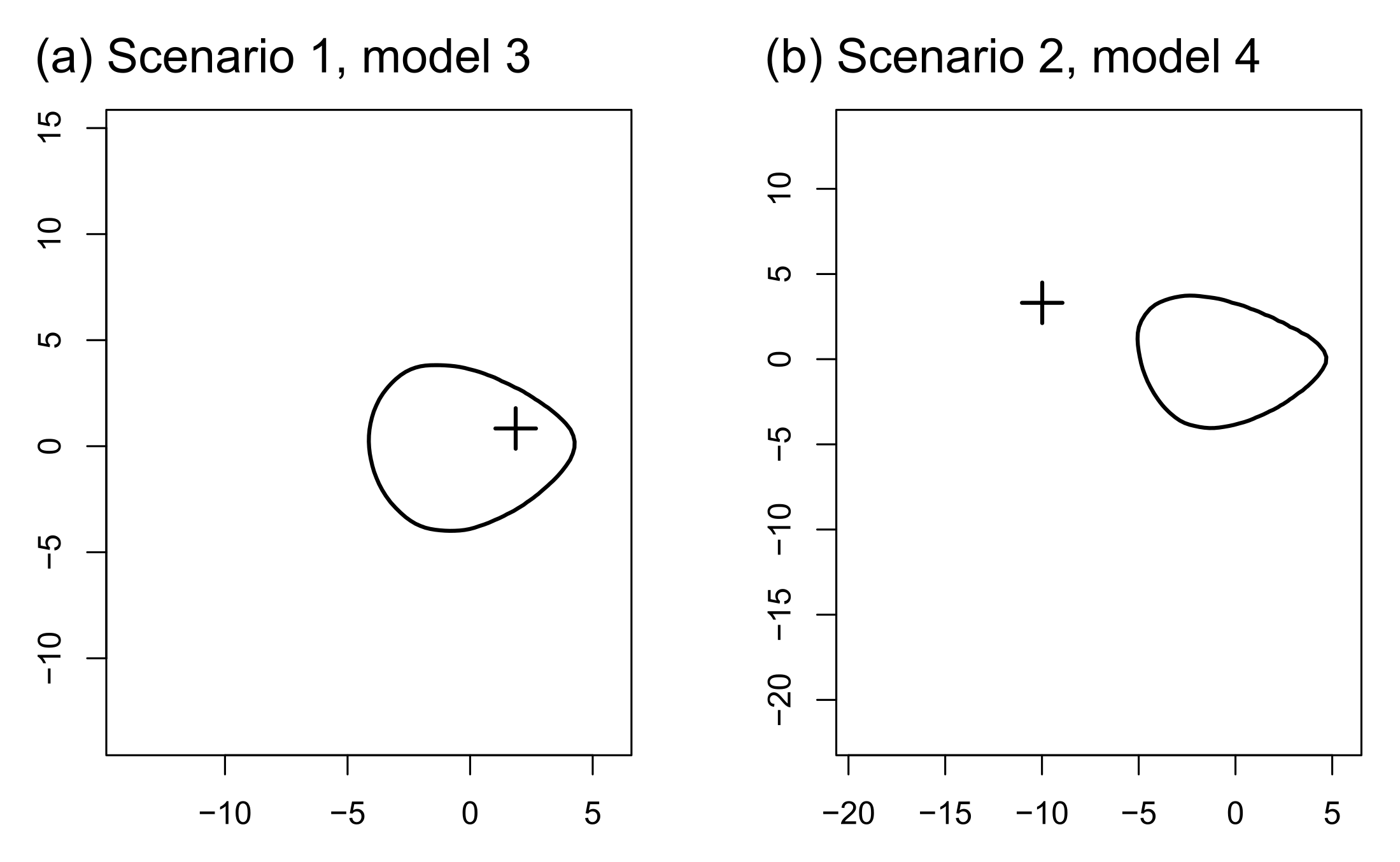

Supplement: Figure S4 — Goodness-of-fit PCA analysis of the summary statistics simulated under Model 2. [file Image_4.tif]
