# Supplementary material for: Signature of the Paleo-Course Changes in the São Francisco River as Source of Genetic Structure in Neotropical Pithecopus nordestinus (Phyllomedusinae, Anura) Treefrog
Source: Front Genet. 2019 Aug 14;10:728. doi: 10.3389/fgene.2019.00728 (PMC6702341; doi:10.3389/fgene.2019.00728)
Supplement: Supplementary file 5 [file Table_1.docx]

**S1a:** Specimen information: code in the map (Fig. 3 and Fig.S1), collecting localities and voucher specimens deposited in the Coleção de Anfíbios CFBH, Universidade Estadual Paulista-Unesp, Rio Claro, São Paulo, Brazil and the Museu de Zoologia “Prof. Adão José Cardoso” (ZUEC), Universidade Estadual de Campinas- UNICAMP, Campinas, São Paulo, Brazil.

| **Code/Locality** | **State** | **Voucher** |
| --- | --- | --- |
| 1. Ubajara | CE | CFBH5367; CFBH5369; CFBH5464; CFBH5467; CFBH5468 |
| 1. Macaíba | RN | ZUEC19820; ZUEC19831 |
| 1. Tibau do Sul | RN | CFBH12429; CFBH12430 |
| 1. São Paulo do Potegi | RN | ZUEC19824; ZUEC19834; ZUEC19833; ZUEC19839; ZUEC19851 |
| 1. Araruna | PB | ZUEC19791; ZUEC19848; ZUEC19793; ZUEC19842; ZUEC19841; ZUEC19788; ZUEC19844 |
| 1. Mamanguape | PB | ZUEC19747; ZUEC19748; ZUEC19751; ZUEC19753; ZUEC19754; ZUEC19755; ZUEC19756 |
| 1. João Pessoa | PB | ZUEC19746; ZUEC19743; ZUEC19737; ZUEC19738; ZUEC19739 |
| 1. Cabaceiras | PB | ZUEC19689; ZUEC19691; ZUEC19699; ZUEC19698; ZUEC19702; ZUEC19708; ZUEC19711 |
| 1. Campina Grande | PB | ZUEC19713; ZUEC19717; ZUEC19718; ZUEC19722; ZUEC19724; ZUEC19729; ZUEC19734 |
| 1. Limoeiro | PE | ZUEC19680; ZUEC19661; ZUEC19670; ZUEC19674; ZUEC19686; ZUEC19673; ZUEC19875; ZUEC19685; ZUEC19672; ZUEC19684 |
| 1. Recife | PE | ZUEC19657; ZUEC19658; ZUEC19659 |
| 1. Sanharó | PE | CFBH9438 |
| 1. Bonito | PE | CFBH9414 |
| 1. Poção | PE | ZUEC19634; ZUEC19638; ZUEC1642; ZUEC19644; ZUEC19648; ZUEC19650 |
| 1. Bom Conselho | PE | ZUEC19617; ZUEC19619; ZUEC19622; ZUEC1623; ZUEC19625; ZUEC19629; ZUEC19632; ZUEC19633 |
| 1. São Miguel dos Milagres | AL | ZUEC19597; ZUEC19599; ZUEC19603; ZUEC19604 |
| 1. Caruaru | PE | ZUEC19608; ZUEC19610; ZUEC19612; ZUEC19614; ZUEC19615 |
| 1. Passos do Camaragibe | AL | CFBH1142; CFBH1143 |
| 1. Pilar | AL | ZUEC19576; ZUEC19584; ZUEC19588; ZUEC19592 |
| 1. Satuba | AL | ZUEC18627; ZUEC18633; ZUEC18634; ZUEC18638; ZUEC18640 |
| 1. Rio Largo | AL | ZUEC19773; ZUEC19781; ZUEC19783 |
| 1. São Miguel dos Campos | AL | ZUEC19565-19571 |
| 1. Couripe | AL | CFBH7350 |
| 1. Laranjeiras | SE | ZUEC19895; ZUEC19896; ZUEC19900 |
| 1. Areia Branca | SE | ZUEC19884; ZUEC19901; ZUEC19906 |
| 1. Itabaiana | SE | CFBH3541; CFBH3542 |
| 1. Alagoinhas | BA | ZUEC17832; ZUEC17833 |
| 1. Mata de São João | BA | CFBH13503 |
| 1. Maracás | BA | CFBH9391; CFBH9395 |
| 1. Gandú | BA | CFBH13484 |
| 1. Jequié | BA | CFBH13598; CFBH13606 |
| 1. Aurelino Leal | BA | CFBH9246; CFBH9247 |
| 1. Bom Jesus da Lapa | BA | CFBH11081 |
| 1. Caetité | BA | CFBH11042; CFBH11053; CFBH11094 |

**S1b:** Primers used for amplification and sequencing of mitochondrial and nuclear fragments in *Pithecopus* species.

| **Fragment** | **Primer** | | **Sequências (5’⭢3’)** | **PCR conditions** | | **References** |
| --- | --- | --- | --- | --- | --- | --- |
| **16S¹** | 12sL13  16sBR | TTAGAAGAGGCAAGTCGTAACATGGTA  CCGGTCTGAACTCAGATCACGT | | | 94°C (5’) [94°C (1’) 51,5°C (1’), 72°C (2”30’) x 39], 72°C (8’) | Feller and Hedges, 1998  Palumbi et al1991 |
| **ND2** | ND2-B1  MVZ38 | GCTAACAAAGCTATCGGGCCCAT  TTCTTAGGGCTTTGAAGGCTC | | | 94°C (5’) [94°C (2’30”) 50°C (1’), 72°C (5’) x 35], 72°C (8’) | Carnaval et al 2009 |
| **Rhod** | Rhod1A  Rhod1D | ACCATGAACGGAACAGAAGGYCC  GTAGCGAAGAARCTTCAAMGTA | | | 95°C (5’) [94°C (1’) 55°C (30”), 72°C (2’30) x 39], 72°C (8’) | Bossuyt & Milinkovitch 2000 |
| **SiaH** | Sia1  Sia2 | TCGAGTGCCCCGTGTGYTTYGAYTA  GAAGTGGAAGCCGAAGCAGSWYTGCATCAT | | | 94°C (5’) [94°C (30”) 60°C (30”), 72°C (45”) x 35], 72°C (8’) | Bonacum et al 2001 |

**¹** For sequencing of mitochondrial 16S ribosomal gene we using the internal primers: Hedges 16L2a, Hedges 16H10, 16Sar-L and 16Sbr-H. For primer sequences, see Goebel et al 1999.

**References**

Bonacum J, DeSalle R, O’Grady PO, Oliveira D, Wintermute J, Zilversmith M (2001) New nuclear and mitochondrial primers for systematics and comparative genomics in drosophilidae. Drosophila Information Service, **84**, 201-204.

Bossuyt F, Milinkovitch MC (2000) Convergence adaptative radiations in Madagascar and Asian ranid frogs reveal covariation between larval and adult traits. Proceedings of the National Academy of Sciences USA, **97**, 6585-6590.

Carnaval AC, Hickerson MJ, Haddad CFB, Rodrigues MT, Moritz C (2009) Stability predicts genetic diversity in the Brazilian Atlantic forest hotspot. Science, **323**, 785–789.

Feller A, Hedges SB (1998) Molecular evidence for the early history of living amphibians. Molecular Phylogenetics and Evolution, **9**, 509-516.

Goebel AM, Donnelly JM, Atz ME (1999) PCR primers and amplification methods for 12S ribosomal DNA, the control region, cytochrome oxidase I, and cytochrome b in bufonids and other frogs, and an overview of PCR primers which have amplified DNA in amphibians successfully. Molecular Phylogenetics and Evolution, **11**, 163–199.

Palumi SR, Martin A, McMillan WO, Stice L, Grabowski G (1991) The simple fool’s guide to PCR, version 2.0. Privately published.
