# Supplementary material for: Signature of the Paleo-Course Changes in the São Francisco River as Source of Genetic Structure in Neotropical Pithecopus nordestinus (Phyllomedusinae, Anura) Treefrog
Source: Front Genet. 2019 Aug 14;10:728. doi: 10.3389/fgene.2019.00728 (PMC6702341; doi:10.3389/fgene.2019.00728)
Supplement: Supplementary file 6 [file Table_2.docx]

**Supplemental information:** Ecological niche modeling (ENMs)

**Table S2.** The occurrence points used to infer the potential geographical distribution of *Phitecopus nordestinus.*

| **Specie** | **Longitude** | **Latitude** | **Specie** | **Longitude** | **Latitude** |
| --- | --- | --- | --- | --- | --- |
| *Phitecopus nordestinus* | -43.9406 | -4.25528 | *Phitecopus nordestinus* | -37.2797 | -7.02444 |
| *Phitecopus nordestinus* | -43.4188 | -13.2546 | *Phitecopus nordestinus* | -37.17 | -10.8064 |
| *Phitecopus nordestinus* | -43.4181 | -13.255 | *Phitecopus nordestinus* | -37.1052 | -10.4793 |
| *Phitecopus nordestinus* | -42.8192 | -14.9261 | *Phitecopus nordestinus* | -37.0241 | -10.5841 |
| *Phitecopus nordestinus* | -42.6992 | -9.01556 | *Phitecopus nordestinus* | -36.705 | -8.18639 |
| *Phitecopus nordestinus* | -42.5069 | -11.8546 | *Phitecopus nordestinus* | -36.6811 | -9.17056 |
| *Phitecopus nordestinus* | -42.4862 | -14.0648 | *Phitecopus nordestinus* | -36.6797 | -9.16972 |
| *Phitecopus nordestinus* | -42.475 | -14.0694 | *Phitecopus nordestinus* | -36.6611 | -9.7525 |
| *Phitecopus nordestinus* | -42.4333 | -14.8497 | *Phitecopus nordestinus* | -36.5864 | -10.2903 |
| *Phitecopus nordestinus* | -41.8556 | -11.3044 | *Phitecopus nordestinus* | -36.5656 | -8.36056 |
| *Phitecopus nordestinus* | -41.817 | -2.83678 | *Phitecopus nordestinus* | -36.5552 | -8.36238 |
| *Phitecopus nordestinus* | -41.7767 | -2.90472 | *Phitecopus nordestinus* | -36.4093 | -8.12646 |
| *Phitecopus nordestinus* | -41.4586 | -4.42472 | *Phitecopus nordestinus* | -36.4087 | -9.10232 |
| *Phitecopus nordestinus* | -41.0426 | -13.7621 | *Phitecopus nordestinus* | -36.4073 | -9.10309 |
| *Phitecopus nordestinus* | -40.9211 | -3.85444 | *Phitecopus nordestinus* | -36.3508 | -9.78194 |
| *Phitecopus nordestinus* | -40.8394 | -14.8661 | *Phitecopus nordestinus* | -36.2872 | -7.48889 |
| *Phitecopus nordestinus* | -40.4372 | -13.4287 | *Phitecopus nordestinus* | -36.1752 | -7.29348 |
| *Phitecopus nordestinus* | -40.4308 | -13.4411 | *Phitecopus nordestinus* | -36.1719 | -7.29364 |
| *Phitecopus nordestinus* | -40.3608 | -10.7417 | *Phitecopus nordestinus* | -36.0936 | -9.78111 |
| *Phitecopus nordestinus* | -40.0836 | -13.8575 | *Phitecopus nordestinus* | -35.9761 | -8.28333 |
| *Phitecopus nordestinus* | -40.0812 | -13.851 | *Phitecopus nordestinus* | -35.9567 | -9.59722 |
| *Phitecopus nordestinus* | -39.7342 | -14.1381 | *Phitecopus nordestinus* | -35.9007 | -9.48632 |
| *Phitecopus nordestinus* | -39.5431 | -15.3797 | *Phitecopus nordestinus* | -35.8811 | -7.23056 |
| *Phitecopus nordestinus* | -39.4958 | -15.4192 | *Phitecopus nordestinus* | -35.8533 | -9.47833 |
| *Phitecopus nordestinus* | -39.4867 | -13.7442 | *Phitecopus nordestinus* | -35.8244 | -9.56333 |
| *Phitecopus nordestinus* | -39.4829 | -13.7507 | *Phitecopus nordestinus* | -35.7417 | -6.55833 |
| *Phitecopus nordestinus* | -39.4086 | -14.3608 | *Phitecopus nordestinus* | -35.7286 | -8.47028 |
| *Phitecopus nordestinus* | -39.3272 | -14.3167 | *Phitecopus nordestinus* | -35.7265 | -8.47038 |
| *Phitecopus nordestinus* | -39.2844 | -14.5931 | *Phitecopus nordestinus* | -35.5349 | -7.17706 |
| *Phitecopus nordestinus* | -38.4192 | -12.1356 | *Phitecopus nordestinus* | -35.4933 | -9.23833 |
| *Phitecopus nordestinus* | -38.2997 | -12.5306 | *Phitecopus nordestinus* | -35.4852 | -9.34073 |
| *Phitecopus nordestinus* | -38.2518 | -12.0619 | *Phitecopus nordestinus* | -35.4549 | -5.54555 |
| *Phitecopus nordestinus* | -37.4253 | -10.685 | *Phitecopus nordestinus* | -35.4503 | -7.87472 |
| *Phitecopus nordestinus* | -37.4247 | -10.6859 | *Phitecopus nordestinus* | -35.4433 | -6.3174 |
| *Phitecopus nordestinus* | -37.3514 | -7.26694 | *Phitecopus nordestinus* | -35.4311 | -6.32534 |
| *Phitecopus nordestinus* | -37.3153 | -10.7578 | *Phitecopus nordestinus* | -35.3539 | -5.85833 |
| *Phitecopus nordestinus* | -37.3089 | -7.1625 | *Phitecopus nordestinus* | -35.1261 | -6.83861 |
| *Phitecopus nordestinus* | -37.3089 | -7.1625 | *Phitecopus nordestinus* | -35.0922 | -8.11861 |
| *Phitecopus nordestinus* | -35.0813 | -6.50305 | *Phitecopus nordestinus* | -34.8808 | -8.05417 |
