# Supplementary material for: Signature of the Paleo-Course Changes in the São Francisco River as Source of Genetic Structure in Neotropical Pithecopus nordestinus (Phyllomedusinae, Anura) Treefrog
Source: Front Genet. 2019 Aug 14;10:728. doi: 10.3389/fgene.2019.00728 (PMC6702341; doi:10.3389/fgene.2019.00728)
Supplement: Supplementary file 7 [file Table_3.docx]

**Supplemental information:** Ecological niche modeling (ENMs)

**Table S3.** The variable selection (factorial analysis) results. The bold variables indicate the selected variables for ENM building. These variables explain 91.3% climate variation of our study area.

| **Variables** | **Factor 1** | **Factor 2** | **Factor 3** | **Factor 4** | **Factor 5** |
| --- | --- | --- | --- | --- | --- |
| Annual Mean Temperature | 0.92 | 0.08 | 0.24 | 0.18 | 0.22 |
| **Mean Diurnal Range** | **0.97** | 0.01 | 0.11 | 0.18 | 0.01 |
| Isothermality | 0.86 | 0.09 | 0.32 | 0.2 | 0.31 |
| Temperature Seasonality | 0.2 | 0.56 | 0.77 | 0.09 | 0.1 |
| Max Temperature of Warmest Month | 0.29 | 0.05 | 0.93 | 0.07 | 0.12 |
| Min Temperature of Coldest Month | 0.06 | 0.96 | 0.1 | 0.19 | 0.1 |
| Temperature Annual Range | 0.2 | 0.87 | 0.01 | 0.15 | 0.16 |
| **Mean Temperature of Wettest Quarter** | 0.29 | 0.08 | **0.94** | 0.05 | 0.11 |
| **Mean Temperature of Driest Quarter** | 0.06 | **0.96** | 0.13 | 0.19 | 0.09 |
| Mean Temperature of Warmest Quarter | 0.36 | 0.43 | 0.23 | 0.3 | 0.04 |
| Mean Temperature of Coldest Quarter | 0.25 | 0.44 | 0.43 | 0.43 | 0.11 |
| **Annual Precipitation** | 0.1 | 0.36 | 0.04 | **0.92** | 0.02 |
| **Precipitation of Wettest Month** | 0.53 | 0.13 | 0.29 | 0.26 | **0.72** |
| Precipitation of Driest Month | 0.61 | 0.13 | 0.43 | 0.18 | 0.54 |
| Precipitation Seasonality | 0.91 | 0.19 | 0.16 | 0.27 | 0.12 |
| Precipitation of Wettest Quarter | 0.78 | 0.09 | 0.23 | 0.49 | 0.3 |
| Precipitation of Driest Quarter | 0.39 | 0.22 | 0.18 | 0.76 | 0.44 |
| Precipitation of Warmest Quarter | 0.84 | 0.13 | 0.16 | 0.09 | 0.2 |
| Precipitation of Coldest Quarter | 0.85 | 0 | 0.26 | 0.28 | 0.22 |
| SS loadings | 6.625 | 3.606 | 3.268 | 2.404 | 1.435 |
| Proportion Variation | 0.349 | 0.190 | 0.172 | 0.127 | 0.076 |
| Cumulative Variation | 0.349 | 0.539 | 0.711 | 0.837 | **0.913** |
