# Supplementary material for: Signature of the Paleo-Course Changes in the São Francisco River as Source of Genetic Structure in Neotropical Pithecopus nordestinus (Phyllomedusinae, Anura) Treefrog
Source: Front Genet. 2019 Aug 14;10:728. doi: 10.3389/fgene.2019.00728 (PMC6702341; doi:10.3389/fgene.2019.00728)
Supplement: Supplementary file 8 [file Table_4.docx]

Table S14. Parameters’ prior distributions used in data simulations. For more details, see *ms* documentation (available at http://home.uchicago.edu/rhudson1/source/mksamples.html).

| Parameter | Prior uniform distribution | Prior uniform distribution after parameter restriction step |
| --- | --- | --- |
| N_e_^1^ | 2,500,000 – 9,000,000 | 2,500,000 – 4,000,000 |
| 16S mutation rate^2^ | 2 x 10^-9^ – 5 x 10^-9^ | - |
| ND2 mutation rate | 5 x 10^-9^ – 5 x 10^-8^ | - |
| SiaH mutation rate | 3 x 10^-10^ – 1.5 x 10^-9^ | - |
| Rhodopsin mutation rate | 1 x 10^-10^ – 1 x 10^-9^ | - |
| T1 (Mya) – scenario 1 (Plio-Pleistocene)^3^ | 0.12 – 5.333 | 2 – 5.333 |
| T2 (Mya) – scenario 1 (more recent colonization) | 0 – T1 | - |
| T1 (Mya) – scenario 2 (present-LIG) | 0 – 0.12 | 0.01 – 0.12 |
| T2 (Mya) – scenario 2 (LGM-LIG) | 0.021 – 0.12 | - |
| T3 (Mya) – scenario 2 (present-LGM) | 0 – 0.021 | 0.005 – 0.021 |
| Population bottlenecks^4^ | 0.01 – 0.2 | - |
| Exponential growth (α)^5^ | 0.25 – 0.9 | - |

^1^Effective population size prior distribution was based on information from the mitochondrial loci [16S and ND2 substitution rates (0.28% and 0.957% per million years, respectively) and theta intervals calculated by dnasp (16S = 9–24; ND2 = 21–52)].

^2^Mutation rates based on the 95% HPD obtained in *BEAST analysis for each region.

^3^Divergence times (in million years). For data simulations, values were converted in coalescent units (τ = divergence time (in years)/generation length*4N_e_)

^4^Bottleneck intensity measured as the ratio of the source population (1 to 20%).

^5^ α = [-(1/τ)*log(N_e_ before/ N_e_ after expansion)]. The interval represents a 10 to 75% of population growth since the time of expansion.
