# Supplementary material for: Signature of the Paleo-Course Changes in the São Francisco River as Source of Genetic Structure in Neotropical Pithecopus nordestinus (Phyllomedusinae, Anura) Treefrog
Source: Front Genet. 2019 Aug 14;10:728. doi: 10.3389/fgene.2019.00728 (PMC6702341; doi:10.3389/fgene.2019.00728)
Supplement: Supplementary file 17 [file Table_13.docx]

**Table S13.** Diversity indices estimated for each *P. nordestinus* population. N = number of sequences; h = number of haplotypes; *S* = number of segregating sites; Hd = haplotype diversity; π = nucleotide diversity.

| **Location** | **16S** | | | | | **ND2** | | | | | **SiaH** | | | | | **Rhodopsin** | | | | |
| --- | --- | --- | --- | --- | --- | --- | --- | --- | --- | --- | --- | --- | --- | --- | --- | --- | --- | --- | --- | --- |
|  | **N** | **h** | ***S*** | **Hd (s.d.)** | **π (s.d.)** | **N** | **h** | ***S*** | **Hd (s.d.)** | **π (s.d.)** | **N** | **h** | ***S*** | **Hd (s.d.)** | **π (s.d.)** | **N** | **h** | ***S*** | **Hd (s.d.)** | **π (s.d.)** |
| 1. Ubajara/CE | 3 | 3 | 9 | 1 (0.27) | 0.006 (0.005) | 8 | 3 | 4 | 0.61 (0.16) | 0.002 (0.002) | 4 | 3 | 2 | 0.83 (0.22) | 0.003 (0.003) | 6 | 3 | 2 | 0.73 (0.15) | 0.003 (0.003) |
| 2. Macaiba/RN |  |  |  |  |  | 7 | 5 | 17 | 0.90 (0.1) | 0.008 (0.005) | 26 | 3 | 2 | 0.28 (0.11) | 0.001(0.001) | 22 | 3 | 2 | 0.45 (0.11) | 0.002 (0.002) |
| 3. Tibau do Sul/RN | 2 | 1 | 0 | 0 (0) | 0 (0) | 1 | 1 | 0 | 0 (0) | 0 (0) | 2 | 2 | 2 | 1 (0.5) | 0.005 (0.007) | 4 | 2 | 1 | 0.5 (0.26) | 0.002 (0.002) |
| 4.São Paulo do Potegi/RN | 6 | 5 | 8 | 0.93 (0.12) | 0.003 (0.002) | 6 | 5 | 16 | 0.93 (0.12) | 0.007 (0.005) | 8 | 2 | 1 | 0.25 (0.18) | 0.001 (0.001) | 10 | 3 | 2 | 0.6 (0.13) | 0.002 (0.002) |
| 5.Araruna/PB | 11 | 7 | 15 | 0.87 (0.09) | 0.004 (0.002) | 9 | 3 | 11 | 0.42 (0.19) | 0.003 (0.002) | 12 | 4 | 3 | 0.65 (0.13) | 0.002 (0.002) | 16 | 2 | 1 | 0.46 (0.09) | 0.002 (0.002) |
| 6. Mamanguape/PB | 7 | 3 | 5 | 0.67 (0.16) | 0.002 (0.002) | 9 | 2 | 3 | 0.22 (0.17) | 0.001 (0.001) | 20 | 4 | 3 | 0.44 (0.13) | 0.001 (0.001) | 14 | 2 | 1 | 0.49 (0.09) | 0.002 (0.002) |
| 7. João Pessoa/PB | 8 | 2 | 1 | 0.25 (0.18) | 0 (0) | 6 | 2 | 6 | 0.33 (0.21) | 0.002 (0.002) | 20 | 3 | 2 | 0.28 (0.12) | 0.001 (0.001) | 14 | 3 | 2 | 0.47 (0.14) | 0.002 (0.002) |
| 8. Cabaceiras/PB | 11 | 6 | 8 | 0.87 (0.07) | 0.003 (0.002) | 8 | 5 | 13 | 0.79 (0.15) | 0.006 (0.004) | 24 | 5 | 4 | 0.38 (0.12) | 0.001 (0.001) | 18 | 3 | 3 | 0.63 (0.06) | 0.003 (0.003) |
| 9. Campina Grande/PB | 11 | 3 | 5 | 0.56 (0.13) | 0.002 (0.001) | 10 | 3 | 9 | 0.51 (0.16) | 0.004 (0.002) | 26 | 4 | 3 | 0.56 (0.08) | 0.002 (0.002) | 20 | 4 | 2 | 0.62 (0.06) | 0.002 (0.002) |
| 10.Limoeiro/PE | 10 | 2 | 1 | 0.2 (0.15) | 0 (0) | 15 | 2 | 1 | 0.13 (0.11) | 0 (0) | 24 | 3 | 2 | 0.56 (0.07) | 0.002 (0.002) | 18 | 4 | 2 | 0.47 (0.13) | 0.002 (0.002) |
| 11. Recife/PE | 5 | 2 | 1 | 0.4 (0.24) | 0 (0) | 5 | 1 | 0 | 0 (0) | 0 (0) | 2 | 2 | 1 | 1 (0.5) | 0.003 (0.004) | 8 | 2 | 1 | 0.54 (0.12) | 0.002 (0.002) |
| 12. Sanharó/PE | 2 | 2 | 1 | 1 (0.5) | 0.001 (0.001) |  |  |  |  |  | 4 | 2 | 1 | 0.5 (0.26) | 0.001 (0.002) | 4 | 2 | 1 | 0.5 (0.26) | 0.002 (0.002) |
| 13. Bonito/PE |  |  |  |  |  | 1 | 1 | 0 | 0 (0) | 0 (0) | 2 | 2 | 2 | 1 (0.5) | 0.005 (0.007) |  |  |  |  |  |
| 14. Poção/PE | 7 | 3 | 4 | 0.76 (0.11) | 0.002 (0.001) | 7 | 4 | 10 | 0.86 (0.1) | 0.006 (0.004) | 4 | 2 | 1 | 0.5 (0.26) | 0.001 (0.002) | 16 | 4 | 3 | 0.62 (0.1) | 0.003 (0.002) |
| 15. Bom Conselho/PE | 10 | 4 | 7 | 0.53 (0.18) | 0.002 (0.001) | 8 | 4 | 3 | 0.75 (0.14) | 0.001 (0.001) | 6 | 1 | 0 | 0 (0) | 0 (0) | 20 | 3 | 2 | 0.47 (0.1) | 0.002 (0.002) |
| 16. São Miguel dos Milagres/AL | 11 | 1 | 0 | 0 (0) | 0 (0) | 4 | 1 | 0 | 0 (0) | 0 (0) | 14 | 2 | 1 | 0.26 (0.14) | 0.001 (0.001) | 20 | 2 | 1 | 0.34 (0.11) | 0.001 (0.001) |
| 17. Caruaru/PE | 6 | 1 | 0 | 0 (0) | 0 (0) | 5 | 3 | 2 | 0.7 (0.22) | 0.001 (0.001) | 6 | 1 | 0 | 0 (0) | 0 (0) | 14 | 3 | 2 | 0.66 (0.07) | 0.003 (0.002) |
| 18.Passo Camarajibe/AL | 3 | 2 | 1 | 0.67 (0.31) | 0.001 (0.001) | 2 | 2 | 3 | 1 (0.5) | 0.004 (0.004) | 4 | 2 | 1 | 0.5 (0.26) | 0.001 (0.002) | 2 | 1 | 0 | 0 (0) | 0 (0) |
| 19. Pilar/AL | 5 | 1 | 0 | 0 (0) | 0 (0) | 8 | 1 | 0 | 0 (0) | 0 (0) | 16 | 3 | 2 | 0.24 (0.13) | 0.001 (0.001) | 20 | 3 | 2 | 0.59 (0.07) | 0.002 (0.002) |
| 20. Satuba/AL | 10 | 2 | 1 | 0.47 (0.13) | 0 (0) | 6 | 3 | 4 | 0.73 (0.15) | 0.002 (0.002) | 14 | 3 | 2 | 0.27 (0.15) | 0.001 (0.001) | 20 | 3 | 2 | 0.28 (0.12) | 0.001 (0.001) |
| 21.Rio Largo/AL |  |  |  |  |  | 1 | 1 | 0 | 0 (0) | 0 (0) | 10 | 2 | 1 | 0.2 (0.15) | 0.001 (0.001) | 12 | 2 | 1 | 0.17 (0.13) | 0.001 (0.001) |
| 22. São Miguel dos Campos/AL | 1 | 1 | 0 | 0 (0) | 0 (0) | 5 | 5 | 8 | 1 (0.13) | 0.005 (0.003) | 10 | 2 | 1 | 0.36 (0.16) | 0.001 (0.001) | 12 | 3 | 2 | 0.53 (0.14) | 0.002 (0.002) |
| 23. Couripe/AL |  |  |  |  |  |  |  |  |  |  | 2 | 1 | 0 | 0 (0) | 0 (0) | 2 | 2 | 1 | 1 (0.5) | 0.003 (0.005) |
| 24. Laranjeiras/SE | 3 | 2 | 2 | 0.67 (0.31) | 0.001 (0.001) | 7 | 2 | 14 | 0.47 (0.17) | 0.008 (0.005) | 6 | 5 | 7 | 0.93 (0.12) | 0.008 (0.006) | 22 | 3 | 2 | 0.58 (0.08) | 0.002 (0.002) |
| 25. Areia Branca/SE | 2 | 1 | 0 | 0 (0) | 0 (0) | 9 | 5 | 19 | 0.72 (0.16) | 0.009 (0.005) | 22 | 11 | 8 | 0.88 (0.05) | 0.009 (0.005) | 36 | 4 | 3 | 0.54 (0.06) | 0.002 (0.002) |
| 26. Itabaiana/SE | 2 | 2 | 4 | 1 (0.5) | 0.004 (0.004) | 2 | 2 | 12 | 1 (0.5) | 0.015 (0.015) |  |  |  |  |  |  |  |  |  |  |
| 27. Alagoinhas/BA | 3 | 3 | 5 | 1 (0.27) | 0.003 (0.003) | 4 | 3 | 10 | 0.83 (0.22) | 0.006 (0.004) | 6 | 2 | 2 | 0.6 (0.13) | 0.003 (0.003) | 10 | 3 | 2 | 0.69 (0.1) | 0.003 (0.002) |
| 28. Mata de São João/BA | 1 | 1 | 0 | 0 (0) | 0 (0) | 1 | 1 | 0 | 0 (0) | 0 (0) | 2 | 2 | 1 | 1 (0.5) | 0.003 (0.004) |  |  |  |  |  |
| 29. Maracás/BA | 2 | 1 | 0 | 0 (0) | 0 (0) | 5 | 4 | 9 | 0.9 (0.16) | 0.005 (0.003) |  |  |  |  |  | 4 | 3 | 2 | 0.83 (0.22) | 0.003 (0.003) |
| 30. Gandú/BA | 1 | 1 | 0 | 0 (0) | 0 (0) | 1 | 1 | 0 | 0 (0) | 0 (0) | 2 | 1 | 0 | 0 (0) | 0 (0) |  |  |  |  |  |
| 31. Jequié/BA | 2 | 2 | 6 | 1 (0.5) | 0.006 (0.006) | 2 | 2 | 10 | 1 (0.5) | 0.012 (0.013) | 4 | 2 | 2 | 0.5 (0.26) | 0.003 (0.003) | 4 | 2 | 1 | 0.67 (0.2) | 0.002 (0.003) |
| 32. Aurelino Leal/BA | 2 | 1 | 0 | 0 (0) | 0 (0) | 3 | 3 | 7 | 1 (0.27) | 0.006 (0.005) | 4 | 3 | 4 | 0.83 (0.22) | 0.006 (0.005) | 6 | 2 | 1 | 0.6 (0.13) | 0.002 (0.002) |
| 33. Bom Jesus da Lapa/BA | 1 | 1 | 0 | 0 (0) | 0 (0) |  |  |  |  |  |  |  |  |  |  | 2 | 2 | 1 | 1 (0.5) | 0.003 (0.005) |
| 34. Caetité/BA | 3 | 3 | 64 | 1 (0.27) | 0.041 (0.031) | 3 | 2 | 92 | 0.67 (0.31) | 0.075 (0.057) | 4 | 2 | 1 | 0.5 (0.26) | 0.001 (0.002) | 2 | 2 | 1 | 1 (0.5) | 0.003 (0.005) |
| Total | 151 | 44 | 107 | 0.89 (0.02) | 0.017 (0.008) | 168 | 62 | 167 | 0.95 (0.01) | 0.042 (0.021) | 310 | 25 | 19 | 0.57 (0.03) | 0.005 (0.003) | 378 | 13 | 10 | 0.57 (0.03) | 0.002 (0.002) |
