# Supplementary material for: Signature of the Paleo-Course Changes in the São Francisco River as Source of Genetic Structure in Neotropical Pithecopus nordestinus (Phyllomedusinae, Anura) Treefrog
Source: Front Genet. 2019 Aug 14;10:728. doi: 10.3389/fgene.2019.00728 (PMC6702341; doi:10.3389/fgene.2019.00728)
Supplement: Supplementary file 18 [file Table_14.docx]

**Table S14.** Neutrality tests' results for each *P. nordestinus* population. *D* = Tajima's D; *Fs* = Fu's Fs; *R2* = Rozas & Ramos-Onsis' R2. In bold, the statistical significant values (*p<0.05, **p<0.02).

| Location | **16S** | | | **ND2** | | | **SiaH** | | | **Rhodopsin** | | |
| --- | --- | --- | --- | --- | --- | --- | --- | --- | --- | --- | --- | --- |
|  | *D* | *Fs* | *R2* | *D* | *Fs* | *R2* | *D* | *Fs* | *R2* | *D* | *Fs* | *R2* |
| Ubajara/CE | 0,00 | 0,59 | 0,47 | 0,89 | 1,41 | 0,22 | -0,71 | -0,89 | 0,25 | 0,31 | -0,30 | 0,24 |
| Macaiba/RN |  |  |  | -0,29 | 0,83 | 0,17 | -0,93 | -1,01 | **0.07**** | -0,23 | -0,17 | 0,12 |
| Tibau do Sul/RN | 0,00 | 0,00 | 0,00 | 0,00 | 0,00 | 0,00 | 0,00 | 0,69 | 0,50 | -0,61 | 0,17 | 0,43 |
| São Paulo do Potegi/RN | -1,07 | -1,37 | 0,22 | -0,89 | -0,04 | 0,19 | -1,05 | -0,18 | 0,33 | -0,18 | -0,27 | 0,19 |
| Araruna/PB | -1,00 | -0,93 | 0,12 | **-1.67*** | 2,46 | 0,19 | -0,73 | -1,17 | 0,14 | 1,03 | 1,10 | 0,23 |
| Mamanguape/PB | 0,59 | 1,60 | 0,21 | **-1.51*** | 1,32 | 0,31 | -1,16 | -1,66 | 0,10 | 1,21 | 1,14 | 0,25 |
| João Pessoa/PB | -1,05 | -0,18 | 0,33 | **-1.37*** | 3,00 | 0,37 | -1,14 | -1,21 | 0,12 | -0,53 | -0,47 | 0,16 |
| Cabaceiras/PB | 0,32 | -0,58 | 0,18 | -0,05 | 0,78 | 0,18 | **-1.69*** | **-3.34**** | 0,08 | 0,22 | 0,98 | 0,16 |
| Campina Grande/PB | 1,02 | 2,06 | 0,22 | 0,12 | 3,36 | 0,17 | -0,48 | -0,79 | 0,11 | 0,63 | -0,76 | 0,20 |
| Limoeiro/PE | 0,00 | -0,34 | 0,00 | -1,16 | -0,65 | 0,25 | 0,36 | 0,35 | 0,16 | -0,03 | -1,36 | 0,14 |
| Recife/PE | -0,82 | 0,09 | 0,40 | 0,00 | 0,00 | 0,00 | 0,00 | 0,00 | 0,50 | 1,17 | 0,87 | 0,27 |
| Sanharó/PE | 0,00 | 0,00 | 0,50 |  |  |  | -0,61 | 0,17 | 0,43 | -0,61 | 0,17 | 0,43 |
| Bonito/PE |  |  |  | 0,00 | 0,00 | 0,00 | 0,00 | 0,69 | 0,50 |  |  |  |
| Poção/PE | 1,46 | 1,51 | 0,27 | 1,27 | 1,75 | 0,25 | -0,61 | 0,17 | 0,43 | -0,41 | -0,82 | 0,14 |
| Bom Conselho/PE | -1,49 | 0,05 | 0,16 | -0,81 | -1,39 | 0,16 | 0,00 | 0,00 | 0,00 | -0,29 | -0,23 | 0,15 |
| São Miguel dos Milagres/AL | 0,00 | 0,00 | 0,00 | 0,00 | 0,00 | 0,00 | -0,34 | 0,19 | **0.13*** | 0,35 | 0,72 | 0,17 |
| Caruaru/PE | 0,00 | 0,00 | 0,00 | 0,24 | -0,48 | 0,25 | 0,00 | 0,00 | 0,00 | 0,70 | 0,38 | 0,20 |
| Passo de Camarajibe/AL | 0,00 | 0,20 | 0,50 | 0,00 | 1,10 | 0,50 | -0,61 | 0,17 | 0,43 | 0,00 | 0,00 | 0,00 |
| Pilar/AL | 0,00 | 0,00 | 0,00 | 0,00 | 0,00 | 0,00 | -1,50 | **-1.61*** | 0,16 | 0,44 | 0,35 | 0,17 |
| Satuba/AL | 0,82 | 0,82 | 0,23 | 0,56 | 0,96 | 0,22 | **-1.48*** | **-1.47*** | 0,17 | -1,14 | -1,21 | 0,12 |
| Rio Largo/AL |  |  |  | 0,00 | 0,00 | 0,00 | -1,11 | -0,34 | 0,30 | -1,14 | -0,48 | 0,28 |
| São Miguel dos Campos/AL | 0,00 | 0,00 | 0,00 | -0,07 | -1,80 | 0,18 | 0,01 | 0,42 | 0,18 | -0,38 | -0,36 | 0,17 |
| Couripe/AL |  |  |  |  |  |  | 0,00 | 0,00 | 0,00 | 0,00 | 0,00 | 0,00 |
| Laranjeiras/SE | 0,00 | 1,06 | 0,47 | 0,92 | 7,25 | 0,24 | -0,12 | -1,28 | 0,16 | 0,47 | 0,41 | 0,17 |
| Areia Branca/SE | 0,00 | 0,00 | 0,00 | 0,24 | 2,17 | 0,17 | 1,46 | -3,18 | 0,20 | -0,38 | -0,65 | 0,11 |
| Itabaiana/SE | 0,00 | 1,39 | 0,50 | 0,00 | 2,48 | 0,50 |  |  |  |  |  |  |
| Alagoinhas/BA | 0,00 | -0,08 | 0,24 | -0,83 | 1,51 | 0,38 | 1,75 | 1,94 | 0,30 | 0,53 | 0,11 | 0,21 |
| Mata de São João/BA | 0,00 | 0,00 | 0,00 | 0,00 | 0,00 | 0,00 | 0,00 | 0,00 | 0,00 |  |  |  |
| Maracás/BA | 0,00 | 0,00 | 0,00 | -0,53 | 0,13 | 0,27 |  |  |  | -0,71 | -0,89 | 0,25 |
| Gandú/BA | 0,00 | 0,00 | 0,00 | 0,00 | 0,00 | 0,00 | 0,00 | 0,00 | 0,00 |  |  |  |
| Jequié/BA | 0,00 | 1,79 | 0,50 | 0,00 | 2,30 | 0,50 | -0,71 | 1,10 | 0,43 | 1,63 | 0,54 | 0,33 |
| Aurelino Leal/BA | 0,00 | 0,00 | 0,00 | 0,00 | 0,31 | 0,24 | -0,07 | 0,25 | 0,33 | 1,45 | 0,80 | 0,30 |
| Bom Jesus da Lapa/BA | 0,00 | 0,00 | 0,00 |  |  |  |  |  |  | 0,00 | 0,00 | 0,00 |
| Caetité/BA | 0,00 | 2,64 | 0,46 | 0,00 | 7,59 | 0,47 | -0,61 | 0,17 | 0,43 | 0,00 | 0,00 | 0,00 |
| Total | -0,43 | -1,42 | 0,07 | 0,57 | 0,00 | 0,10 | -1,08 | **-13.47**** | 0,04 | -1,21 | **-7.90**** | 0,03 |
