# Supplementary material for: Signature of the Paleo-Course Changes in the São Francisco River as Source of Genetic Structure in Neotropical Pithecopus nordestinus (Phyllomedusinae, Anura) Treefrog
Source: Front Genet. 2019 Aug 14;10:728. doi: 10.3389/fgene.2019.00728 (PMC6702341; doi:10.3389/fgene.2019.00728)
Supplement: Supplementary file 19 [file Table_15.docx]

**Table S15.** ABC cross-validation analysis’ results based on 100 pseudo-observations for each model (threshold of 5%) showing the number of times the models were recovered in each sample. The values in bold show the models recovered with higher frequency in each evaluation.

|  | Model 1 | Model 2 | Model 3 | Model 4 | Model 5 | Model 6 |
| --- | --- | --- | --- | --- | --- | --- |
| Model 1 | **77** | 13 | 10 | 0 | 0 | 1 |
| Model 2 | 24 | **73** | 3 | 0 | 0 | 0 |
| Model 3 | 28 | 4 | **68** | 0 | 0 | 0 |
| Model 4 | 0 | 0 | 0 | **51** | 15 | 34 |
| Model 5 | 0 | 0 | 0 | **40** | 24 | 36 |
| Model 6 | 0 | 0 | 0 | 31 | 17 | **52** |
